# Toward Reliable Diabetic Retinopathy Screening

**DOI:** 10.3390/s26144515

**Published:** 2026-07-16

**Authors:** Hendrio Bragança, Ítalo P. Caliari, Wington L. Vital, Antonio Fontenele, Sergio Cavalcante, Glaucio Messias

**Affiliations:** Núcleo de Capacitação em Inteligência Artificial (NCIA), Fundação Paulo Feitoza—FPFtech, Av. Danilo de Matos Areosa, 1170, Distrito Industrial, Manaus 69075-351, Amazonas, Brazil; italo.caliari@fpf.br (Í.P.C.); wington.vital@fpf.br (W.L.V.); antonio.fontenele@fpf.br (A.F.); sergio.cavalcante@fpf.br (S.C.); glaucio.messias@fpf.br (G.M.)

**Keywords:** diabetic retinopathy grading, ordinal learning, multi-scale feature fusion, frequency-aware fusion, explainable artificial intelligence

## Abstract

Diabetic retinopathy (DR) grading requires reliable five-grade severity assessment under substantial acquisition variability and cross-dataset distribution shift. We propose PRISM-DR, a multi-objective five-grade DR grading framework trained under a gradient-partitioned strategy. The architecture is organized as a feedforward pipeline: a data-driven preprocessing stage followed by a ConvNeXtV2-Base backbone, a Recurrent BiFPN neck for multi-scale feature fusion, a Frequency-Aware Fusion module, a lightweight multi-scale reasoning transformer, dual classification heads with gradient-isolated pathways (categorical and ordinal), and a prototype memory module for embedding regularization. The CORAL ordinal head operates through a dedicated projection layer and is gradient-isolated from the backbone; the backbone is shaped by the cross-entropy, prototype contrastive, and view-consistency objectives, which carry indirect ordinal signal through severity-weighted class penalties and grade-indexed cluster regularization. The model is trained in a multi-crop setting with a phased loss curriculum designed for severely imbalanced DR datasets. Evaluated across six datasets under Fixed-Source, Multi-Target (FSMT) protocols, PRISM-DR trained on EyePACS + DDR achieves QWK of 0.835 on IDRiD, 0.865 on APTOS2019, and 0.720 on Messidor-2, with in-domain QWK = 0.920 and AUC-PR = 0.941 on EyePACS, outperforming RETFound, RETFound-Green, and MedGemma-4B in AUC-PR across all evaluated datasets. Quantitative interpretability evaluation against 755 expert-annotated lesion images yields 8.0× Energy Ratio Enrichment and a FAF gate retention ratio of 4.4× inside lesion regions, confirming that anatomically plausible spatial priors emerge from grade-level supervision alone, without pixel-level annotation. PRISM-DR establishes a superior accuracy–robustness–capacity trade-off for scalable, automated DR screening.

## 1. Introduction

Diabetic Retinopathy (DR) is a microvascular complication of diabetes mellitus and remains a leading cause of preventable vision loss among working-age adults worldwide [1,2,3,4,5,6]. As the global burden of diabetes rises, projected by the International Diabetes Federation to reach 643 million people by 2030 [3,4,5], health systems face increasing demand for scalable screening and risk stratification. Clinically, DR evolves along a severity continuum from early non-proliferative stages, which may be asymptomatic, to proliferative disease with neovascular complications that can cause irreversible blindness. Because timely intervention is highly effective in preventing visual impairment, early detection and accurate grading are central to screening programs [7].

In routine practice, screening is commonly performed using retinal fundus photographs that are graded by trained clinicians. However, manual grading is labor-intensive and time-consuming, and it is sensitive to inter- and intra-grader variability, particularly at boundaries between adjacent severity levels. These practical constraints become more pronounced as screening coverage expands and specialist availability remains limited, motivating automated approaches that can support consistent, high-throughput assessment at population scale. Deep learning has substantially advanced automated fundus analysis, with landmark studies demonstrating expert-level performance in detecting clinically referable DR [8,9,10]. Most prior work has relied on convolutional backbones fine-tuned from natural-image pretraining [11,12,13]. Despite their success, five-grade DR severity assessment remains challenging for three intertwined reasons. First, diagnostically relevant lesions are often small, sparse, and distributed across the retina, requiring the model to reconcile fine-scale evidence (e.g., microaneurysms and small hemorrhages) with global severity context (e.g., lesion burden and distribution). Second, the ICDR scale is ordinal: errors are not equally harmful, and neighboring grades can be visually similar, which makes conventional flat multi-class objectives misaligned with clinical semantics. Third, fundus images exhibit substantial domain variability due to device-specific color response, illumination, field-of-view, and post-processing pipelines, frequently causing performance degradation under cross-domain deployment.

To address these challenges, we propose PRISM-DR, a multi-objective framework for five-grade DR severity assessment that explicitly targets multi-scale lesion modeling, ordinal-calibrated prediction, and robustness to acquisition shift without requiring pixel-level lesion annotations. The model couples a shared ConvNeXtV2 [14] trunk with a multi-crop forward strategy that processes a global view and multiple local views, encouraging complementary evidence aggregation. To improve domain robustness at the input level, a lightweight Learned Style Adapter (LSA) performs sample-conditioned channel modulation, providing a learnable alternative to fixed color normalization. To capture lesion cues across resolutions, the network taps intermediate backbone features and fuses them with a Recurrent BiFPN neck, which iterates bidirectional top-down and bottom-up fusion passes across three loops using LTI-stable lateral states and fast-normalized fusion weights. We further introduce Frequency-Aware Fusion (FAF) on intermediate pyramid levels to preserve high-frequency lesion signatures while suppressing low-frequency nuisance variation. Building on these multi-scale representations, a Multi-Scale CLS Reasoner aggregates spatial scale tokens via a Recurrent Depth Transformer, producing a compact global embedding for dual-head prediction.

The model employs two output heads with distinct roles and isolated gradient pathways. The cross-entropy head shapes the backbone directly, using severity-weighted class penalties that increase monotonically with ICDR grade, alongside EMA prototype contrastive regularization that enforces grade-indexed cluster separation. The CORAL ordinal head maps the backbone embedding to a monotonicity-constrained severity scale via its own dedicated projection, but is gradient-isolated from the backbone to prevent curvature-induced optimization conflict between the softmax and per-threshold binary cross-entropy objectives. Ordinal structure is thus enforced structurally through monotone threshold biases in the CORAL head and indirectly through the backbone, via severity-weighted CE penalties and prototype regularization. Training operates under a phased loss curriculum that introduces each objective progressively to improve stability on heavily imbalanced DR datasets.

In summary, our contributions are as follows.

- A multi-objective architecture (PRISM-DR) integrating multi-crop learning, a Recurrent BiFPN neck with LTI-stable lateral states, Frequency-Aware Fusion, and a Multi-Scale CLS Reasoner with shared Recurrent Depth Transformer for five-grade DR assessment. The backbone and CORAL head operate under isolated gradient pathways to resolve optimization conflict between losses with differing curvature; ordinal structure reaches the backbone indirectly through severity-weighted cross-entropy penalties and prototype contrastive regularization.
- A domain robustness module (Learned Style Adapter) for sample-conditioned channel normalization, decoupled from fixed preprocessing and jointly trained with the downstream objective to reduce device-induced appearance shift.
- An ordinal-aware prediction strategy via a CORAL head with monotonicity-constrained biases, complemented by EMA prototype memory and multi-crop consistency regularization under a four-phase loss curriculum.
- Built-in explainability without pixel-level supervision: a comprehensive multi-scale interpretability framework that extracts grade-specific evidence from six internal layers, providing anatomically grounded evidence of lesion-focusing behavior trained solely from image-level grades.
- Empirical evaluation under in-domain and Fixed-Source, Multi-Target (FSMT) cross-domain protocols, with strong cross-dataset transfer and AUC-PR gains over foundation baselines including RETFound, RETFound-Green, and MedGemma-4B.

Our objective is to support clinical decision-making by improving the reliability of severity assignment, which is crucial for determining referral urgency and treatment eligibility. In this setting, the clinical relevance of a five-class grading system extends beyond mere detection; it requires a model capable of distinguishing subtle boundaries between adjacent grades (e.g., mild vs. moderate NPDR) while minimizing “large distance” errors, such as missing vision-threatening proliferative disease. Furthermore, because current therapies such as anti-VEGF are most effective when initiated before permanent damage occurs, the integration of automated tools into primary care could facilitate earlier intervention and prevent irreversible vision loss. By providing a scalable and objective means of evaluation, deep learning serves as a critical bridge between the high volume of global diabetes cases and the limited availability of specialized ophthalmic care, ultimately advancing the goal of precision medicine in diabetic eye disease.

## 2. Background

This section introduces the key concepts required to contextualize DR severity grading from retinal fundus photographs. DR is a prevalent and vision-threatening complication of diabetes mellitus, a condition characterized by chronic metabolic stress and hyperglycemia that progressively damage the microvasculature. This systemic vascular dysfunction compromises retinal integrity and contributes to the development and progression of DR.

**Figure 1:**
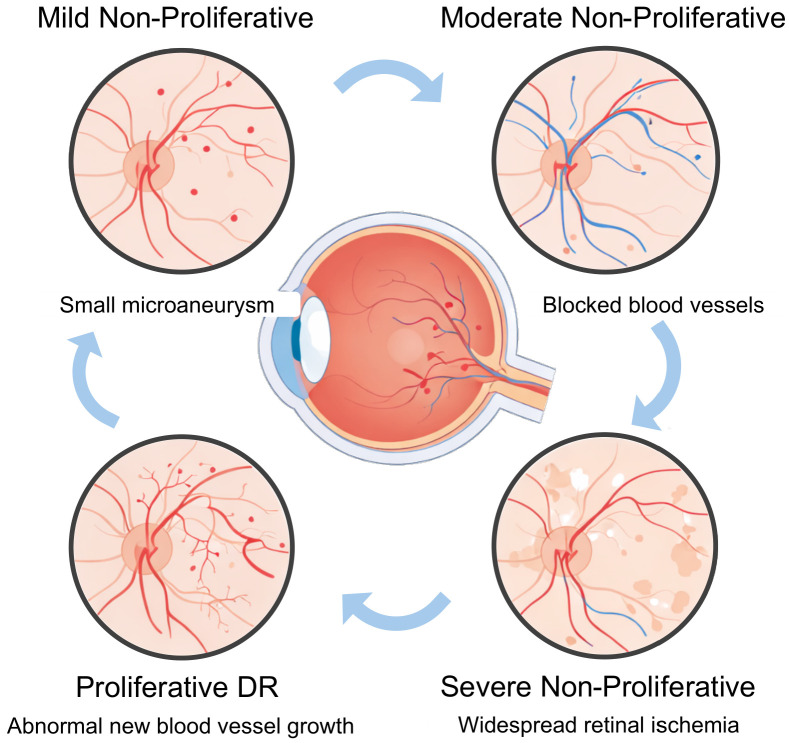
Schematic representation of ICDR diabetic retinopathy severity progression. The figure illustrates representative funduscopic signs associated with increasing DR severity, including early microaneurysms in mild non-proliferative DR, vascular obstruction and hemorrhagic changes in moderate non-proliferative DR, widespread retinal ischemia in severe non-proliferative DR, and abnormal neovascular growth in proliferative DR. These progressive retinal findings motivate the ordered five-grade ICDR scale used for clinical screening and automated severity grading.

In DR, progressive microvascular dysfunction and neuroglial stress compromise retinal integrity. Because early DR is frequently asymptomatic, population-level screening aims to identify disease before irreversible visual loss occurs, prioritize timely referral, and monitor progression under therapy [6,15,16]. In clinical workflows, these decisions are typically guided by standardized severity grading systems, most commonly the International Clinical Diabetic Retinopathy (ICDR) scale [17], as illustrated in Figure 1 and summarized in Table 1. The ICDR scale stratifies disease severity into five ordered categories (0–4) based on funduscopic signs [17,18]. This ordinal staging is clinically meaningful: it reflects increasing risk of complications, informs follow-up intervals, and provides a practical surrogate for underlying ischemic burden and angiogenic activity.

Retinal color fundus photography remains the most widely deployed imaging modality for DR screening due to its low cost, rapid acquisition, and compatibility with teleophthalmology programs. It helps to identify individuals requiring closer monitoring or referral for treatment [6,16]. In this setting, the clinical objective is not only to detect DR but also to determine severity, since management decisions (follow-up interval, referral urgency, and treatment eligibility) depend on disease stage.

Accurate grading is critical because the cost of misclassification is not uniform: confusing adjacent grades may have limited impact on management, whereas large-distance errors (e.g., missing PDR) may delay treatment and substantially increase risk of severe visual impairment [15]. At the same time, the boundaries between mild, moderate, and severe NPDR can be subtle and subject to inter-grader variability, particularly when lesions are sparse, localized, or masked by acquisition artifacts. This combination of ordered labels, fine-grained cues, and clinically asymmetric error costs motivates modeling and evaluation protocols that respect the grading structure.

### 2.1. Pathophysiological Basis of Diabetic Retinopathy

Under physiological conditions, retinal homeostasis is maintained by a tightly regulated blood–retina barrier (BRB) that preserves the neural microenvironment required for phototransduction. The inner BRB is formed by endothelial tight junctions supported by pericytes and glial end-feet, whereas the outer BRB is maintained by the retinal pigment epithelium (RPE), separating the neural retina from the choroidal circulation. DR progressively compromises this coupled neurovascular unit: endothelial dysfunction and pericyte loss weaken capillary walls, while glial dysregulation and inflammatory activation further destabilize barrier function and vascular autoregulation [15].

The earliest clinically observable manifestations of DR arise from focal microvascular weakening and capillary outpouchings. These changes reflect endothelial injury, pericyte dropout, and basement membrane remodelling, which together increase vascular fragility and local permeability. Clinically, microaneurysms are the hallmark lesion of early disease and define mild NPDR in the ICDR scale [17]. Small intraretinal haemorrhages may accompany microaneurysms as disease advances, but early DR can remain subtle and spatially sparse, making detection highly sensitive to image quality and resolution.

**Figure 2:**
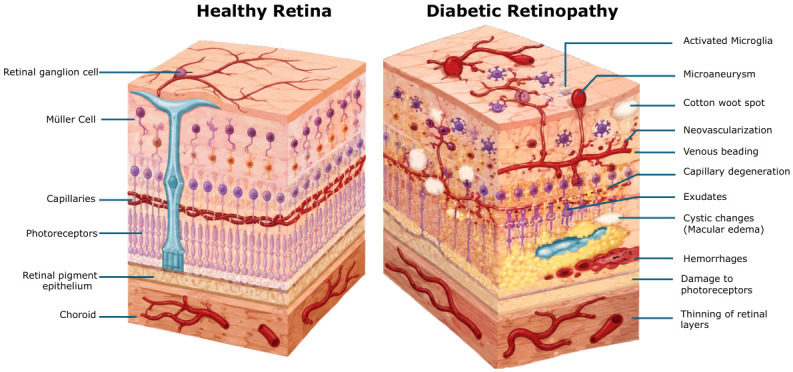
Three-dimensional neurovascular architecture of the human retina in health versus diabetic retinopathy (DR) with diabetic macular edema (DME). ***Left (Healthy Retina):*** Baseline physiological state demonstrating a tightly regulated blood–retina barrier (BRB). Retinal ganglion cells and superficial vessels sit atop organized intermediate layers supported structurally and metabolically by vertical Müller cells, spanning down to the photoreceptor layer (rods and cones), the underlying retinal pigment epithelium (RPE/outer BRB), and the vascular choroid. ***Right (Diabetic Retinopathy):*** Progressive cascade of microvascular breakdown, neuroinflammation, and neural degeneration. Key structural features pointed out from the superficial to the deep layers include: (i) **Vascular Anomalies:** Outpouching microaneurysms, pathological neovascularization branching above the retinal surface, venous beading, and widespread capillary degeneration leading to localized ischemia. (ii) **Neuroinflammatory Responses:** Clusters of activated microglia exhibiting an amoeboid phenotype alongside cotton-wool spots indicative of micro-infarcts in the nerve fiber layer. (iii) **Barrier Failure Components:** Marked accumulation of hard lipid–protein exudates and large extracellular fluid pockets defining cystic changes (macular edema) due to inner/outer BRB disruption. (iv) **Neurodegeneration:** Explicit mechanical and ischemic damage to the photoreceptor layer, culminating in the macro-structural thinning of the overall retinal layers.

Beyond purely vascular changes, DR involves early and sustained neuroinflammatory and glial alterations. As illustrated in the 3D block model (Figure 2), resting microglia transition toward an activated microglia phenotype near the superficial retinal vessels, while Müller cells become stressed and dysfunctional. These changes contribute to inflammatory amplification, impaired metabolic support, and reduced regulation of fluid/ion balance. Importantly, Müller glia are critical for maintaining retinal extracellular homeostasis; their dysfunction can exacerbate vascular leakage and neuronal vulnerability, reinforcing the cycle of barrier failure and tissue injury [15].

Breakdown of the BRB increases vascular permeability and drives accumulation of intraretinal fluid, particularly in the macula. Clinically, this manifests as diabetic macular edema (DME), which can occur at any DR stage. The schematic highlights cystic changes (macular edema) alongside extracellular exudates as representative structural consequences of barrier leakage within the deeper retinal layers (Figure 2). In colour fundus photography, edema is often inferred indirectly through surrogate signs such as hard exudates and macular appearance changes, whereas optical coherence tomography provides a more direct assessment of fluid compartments.

As microvascular injury accumulates, segments of the capillary network undergo capillary degeneration, producing retinal nonperfusion and ischemia. Ischemic stress promotes vascular calibre abnormalities and progressive tissue dysfunction, often accompanied by lesions such as cotton-wool spots (focal nerve fibre layer infarcts), increasing intraretinal haemorrhage burden, venous beading, and intraretinal microvascular abnormalities (IRMA). These findings are characteristic of severe NPDR and signal a high risk of progression to proliferative disease [15,17]. From an image-analysis perspective, this stage requires integrating both local lesions and broader distributional patterns across retinal regions.

When ischemia becomes extensive, pro-angiogenic mediators (notably VEGF) promote the formation of fragile new vessels on the retinal surface. Proliferative DR (PDR) is defined by neovascularization and/or pre-retinal/vitreous haemorrhage [17]. Neovascular complexes are structurally immature and highly prone to bleeding; subsequent fibrovascular proliferation can exert traction on the retina, increasing the risk of tractional retinal detachment and severe, potentially irreversible vision loss [15].

Although clinical grading is driven largely by inner retinal microvascular signs, DR severely damages the deeper neural architecture. Chronic ischemia and barrier failure eventually lead to explicit damage to photoreceptors (rods and cones) and subsequent thinning of retinal layers (Figure 2). These neurodegenerative changes explain why functional visual acuity can permanently decline even after vascular stabilization, and emphasize that deep structural alterations modulate the background textures exploited by automated deep learning models.

The ICDR system operationalises DR severity using fundus-visible signs that reflect the mechanisms shown in Table 1: grade 1 (mild NPDR) corresponds to microaneurysms; grade 2 (moderate NPDR) captures increasing lesion burden without meeting severe criteria; grade 3 (severe NPDR) corresponds to widespread haemorrhages and/or venous beading and/or IRMA consistent with extensive ischemia; and grade 4 (PDR) reflects the angiogenic phase with neovascularization and/or vitreous/pre-retinal haemorrhage [17]. The cross-sectional view in Figure 2 provides an intuitive mechanistic mapping from barrier dysfunction and ischemia to these clinically used categories, while also emphasizing that DME is a permeability-driven complication that may overlay multiple vascular grades.

Because severity grading is anchored in lesion visibility in colour fundus photography, robust screening systems must detect small focal lesions (e.g., microaneurysms) while also capturing global context (lesion burden, distribution, vascular abnormalities, and neovascular patterns) that distinguish advanced NPDR from earlier stages. The mechanisms highlighted in Figure 2 motivate modelling strategies that are simultaneously multi-scale and context-aware, and that explicitly address ambiguity near adjacent grades where the biological transition is gradual rather than discrete.

### 2.2. Core Challenges in Diabetic Retinopathy Grading

Current screening protocols utilize digital fundus photography to grade disease severity according to the ICDR scale [15]. Retinal fundus photography provides a two-dimensional color projection of the posterior pole, capturing the optic disc, macula, vascular arcades, and surrounding retina in a single image.

From an image formation perspective, the retina presents structures with very different spatial scales: as we depict in Figure 1, large features such as the optic disc and major vessels occupy substantial portions of the field, while clinically salient DR lesions (e.g., microaneurysms, small hemorrhages) may be only a few pixels wide at common acquisition resolutions.

This scale disparity creates a fundamental challenge for computational pipelines: severity classification relies on detecting and integrating evidence from small, sparse findings distributed over a large background. In addition, the retinal scene includes physiological variability (disc size and pallor, vessel caliber, pigmentation, choroidal visibility) that is unrelated to DR severity but can change the overall texture and color statistics of the image, complicating model generalization across populations.

Fundus photographs also exhibit characteristic artifacts that are common in real-world screening data and can dominate failure cases if not handled explicitly. Defocus and motion blur reduce the detectability of small lesions; uneven illumination and vignetting can obscure the peripheral retina; specular reflections and dust introduce bright spots; eyelids, lashes, and partial fields lead to occlusions; and poor fixation can shift clinically relevant regions out of frame. These issues interact with device heterogeneity, differences in sensors, lenses, color response, and onboard processing, producing domain shifts that can be as influential as the underlying pathology. As a result, robust DR grading requires not only learning discriminative retinal features, but also accommodating variability in acquisition protocols and image quality, ideally through principled preprocessing, quality control, and training strategies that reduce reliance on spurious cues.

Automated DR grading is challenging because the task combines fine-grained visual recognition with an ordinal clinical scale, under substantial heterogeneity in both pathology and image acquisition. The five severity categories represent a continuum of disease rather than sharply separable visual patterns, and many images contain mixed evidence: small early lesions may coexist with more advanced ischemic signs, while late-stage changes can be localized and partially captured depending on the field of view. In practice, a model must integrate weak, distributed cues across the retina and translate them into a discrete grade, even when the boundary between adjacent categories is ambiguous and subject to expert disagreement. This ambiguity is amplified by the fact that grade definitions depend not only on lesion presence but also on lesion burden and distribution, which are difficult to estimate from a single two-dimensional photograph.

A central difficulty is the scale mismatch between clinically relevant findings and the overall image size. Lesions such as microaneurysms and small intraretinal hemorrhages may occupy a tiny fraction of pixels, while dominant structures (optic disc, major vessels) and background texture contribute strong signals that are not specific to DR severity. Models trained with global supervision can therefore be prone to shortcut learning, overemphasizing correlated but non-causal cues such as illumination patterns, camera signatures, or border artifacts. At the same time, increasing resolution or using patch-based strategies to preserve lesion detail introduces computational costs and design trade-offs: patch sampling may miss rare lesions, while global downsampling can erase them. Effective grading thus requires architectures and training protocols that can capture multi-scale information (global context for staging and local detail for lesion evidence) without introducing brittle dependencies on dataset-specific acquisition characteristics.

Dataset heterogeneity further complicates the problem by inducing domain shift across populations, devices, and labeling protocols. Differences in camera type, color calibration, compression, and cropping produce systematic changes in intensity distributions and texture statistics; differences in screening settings affect the prevalence of poor-quality images; and differences in annotation procedures (single grader vs adjudicated labels) influence label noise and the consistency of grade thresholds. These factors can lead to marked performance degradation when models are evaluated outside the training domain, even when the underlying clinical task is unchanged. Finally, DR datasets are typically imbalanced, with a predominance of no-DR and mild cases and relatively few severe or proliferative cases, making stable learning of minority classes difficult and increasing the risk of clinically consequential errors. Together, these properties make DR grading a demanding benchmark for robust, clinically aligned computer vision models and motivate evaluation protocols that explicitly test cross-dataset generalization and error severity.

Accurate grading is critical because management decisions (ranging from annual monitoring to urgent panretinal photocoagulation or anti-VEGF therapy) are strictly dictated by these severity thresholds. However, the visual manifestations of DR are heterogeneous; lesions such as microaneurysms can be sparse and subtle, while artifacts in fundus imaging can obscure fine details such as neovascularization. The high inter-grader variability and the asymmetric cost of misclassification, where missing PDR poses a far greater risk of irreversible blindness than confusing early NPDR stages, present significant challenges in manual screening. This complexity motivates the development of deep learning systems capable of effectively extracting high-dimensional features from retinal images to support objective and consistent severity classification [15].

### 2.3. Automated DR with Deep Learning

Deep learning methods for DR grading have evolved from convolutional neural networks (CNNs) trained on color fundus photographs to more diverse model families that include modern convolutional backbones, vision transformers, self-supervised foundation models, and hybrid CNN–Transformer architectures [16,18,19,20,21,22,23,24,25,26,27,28]. The central goal of these approaches is to learn discriminative retinal representations that capture both localized lesion evidence and broader disease context from fundus photographs.

Early automated DR systems typically framed severity grading as image-level classification. In this setting, CNNs became the dominant modeling paradigm because they are particularly well suited to retinal image analysis. Their convolutional filters learn local spatial patterns, making them effective at detecting lesion-like structures such as microaneurysms, hemorrhages, hard exudates, cotton-wool spots, and neovascular abnormalities. In addition, the hierarchical nature of CNNs allows shallow layers to capture low-level cues such as edges, color transitions, vessel boundaries, and small texture changes, while deeper layers encode more abstract disease-related patterns and global retinal appearance. This property is especially important for DR, where the final grade is determined not by a single visual cue, but by the type, quantity, distribution, and severity of multiple retinal abnormalities.

Architectures such as VGG [11], ResNet [12], Inception [29], MobileNet [30], and EfficientNet [31] have therefore been widely adopted for DR grading. Residual and densely connected designs improve optimization in deep networks, while efficient architectures reduce computational cost and make deployment more feasible in screening settings. For fundus photographs, CNNs also benefit from useful inductive biases such as locality, weight sharing, and approximate translation equivariance. These biases make CNNs data-efficient and robust for medical imaging tasks in which diagnostic structures may appear in different retinal regions but retain similar local morphology. As a result, CNNs remain strong baselines and practical choices for DR screening, particularly when labeled data are limited or when computational constraints are important.

However, DR severity cues exist at multiple spatial scales. Some signs are highly localized, such as isolated microaneurysms in early non-proliferative DR, whereas others require wider contextual interpretation, such as extensive hemorrhage burden, venous beading, intraretinal microvascular abnormalities, widespread ischemic appearance, or neovascular patterns. This has motivated multi-scale CNN designs using feature pyramids, attention modules, patch-based inference, and high-resolution training. These strategies aim to preserve fine-lesion visibility while still modeling the broader retinal context required for reliable severity grading. In parallel, some approaches incorporate intermediate lesion detection or segmentation objectives, either as auxiliary tasks or as separate stages feeding into a grading head. Although grading remains attractive because it requires only image-level labels, multi-task and multi-stage designs are often motivated by interpretability and by the clinical intuition that DR grades reflect the type, extent, and distribution of lesions.

Transfer learning has also played a central role in DR modeling. Although fundus datasets can be large relative to many medical imaging domains, they remain limited compared with the diversity encountered in real-world screening programs. Labels may also be noisy due to inter-grader variability, differences in grading protocols, and class imbalance. A common strategy is therefore to initialize models from large-scale natural image pretraining and then fine-tune them on fundus photographs. This often improves convergence and generalization, especially when training data are imbalanced or when high-resolution inputs are required to resolve subtle lesions. Nevertheless, the domain gap between natural images and retinal images remains substantial, affecting color statistics, texture distributions, imaging artifacts, and the prevalence of small diagnostic structures. This has motivated domain-adaptive transfer learning, careful augmentation design, staged unfreezing, and normalization strategies that reduce sensitivity to camera-specific appearance.

More recently, Vision Transformers (ViTs) [32] have introduced a complementary paradigm for DR grading. Unlike CNNs, which build representations through local convolutional operations, ViTs divide an image into patches and use self-attention to model relationships among all patches. This global receptive field is useful for DR because clinically relevant evidence may be spatially distributed across the retina. For example, a model may need to jointly consider lesions near the macula, peripheral hemorrhages, vessel abnormalities, optic disc context, and global image quality before assigning a severity grade. Self-attention allows Transformers to relate distant retinal regions directly, making them well suited to capturing long-range dependencies and global disease burden.

Transformers are also attractive because attention weights can, in principle, provide insight into which image regions contribute to a prediction. This is valuable in DR screening, where interpretability and clinical trust are important. In addition, Transformer-based models can naturally support patch-level reasoning, multiple-instance learning, and token aggregation strategies, which align well with the clinical structure of fundus interpretation: local lesions are identified across the image and then integrated into an overall severity assessment. However, ViTs lack some of the inductive biases that make CNNs data-efficient, such as locality and translation equivariance. Consequently, they are often more data-hungry and may overfit when trained from scratch on smaller medical datasets [33].

To address these limitations, self-supervised learning (SSL) has become increasingly important. Self-supervised pretraining exploits large collections of unlabeled retinal images to learn useful representations without manual annotations, which can then be transferred to DR grading with fewer labeled examples [20,34,35]. In fundus imaging, SSL encourages models to learn stable retinal anatomy, vessel topology, optic disc and macular context, lesion-like texture patterns, and image-quality variations. This is particularly relevant for cross-dataset evaluation, where models must remain robust to shifts in camera device, acquisition protocol, preprocessing, population characteristics, and disease prevalence.

Several SSL paradigms have been explored for retinal images, including contrastive learning, masked image reconstruction, and self-distillation methods such as DINO [36,37]. Contrastive methods encourage images or views with related semantic content to have similar representations, masked reconstruction forces the model to infer missing visual information from surrounding context, and self-distillation can produce semantically meaningful patch-level features without labels. These objectives can improve sample efficiency and yield more transferable representations for downstream DR grading.

Overall, CNNs and Transformers offer complementary advantages for automated DR assessment. CNNs provide strong local feature extraction, data efficiency, and robustness for detecting small lesion patterns, whereas Transformers provide global contextual reasoning and flexible patch-level aggregation. Hybrid architectures attempt to combine these strengths by using convolutional layers for low-level retinal feature extraction and Transformer-style attention for long-range lesion–context integration. This combination is especially suitable for DR severity grading, where the model must detect subtle local abnormalities, integrate evidence across multiple retinal regions, preserve ordinal relationships among disease stages, and remain robust under cross-dataset distribution shift.

### 2.4. Related and Recent Work

Recent work on DR grading has increasingly emphasized domain generalization, motivated by the substantial performance degradation observed when models trained on one fundus dataset are evaluated on another. Differences in camera devices, acquisition protocols, illumination, image resolution, preprocessing pipelines, population characteristics, and grading distributions can produce strong domain shifts. As a result, models that perform well under standard in-domain validation may fail to generalize reliably to external screening cohorts. Table 2 summarizes representative methods evaluated under the Leave-One-Domain-Out (LODO) protocol on three commonly used DR benchmarks: APTOS2019, IDRiD, and Messidor. The reported metrics include AUC, Accuracy (ACC), and F1-score, expressed as percentages.

The first group of methods includes conventional empirical risk minimization and generic domain generalization strategies such as ERM, Mixup, MixStyle, Fishr, and DDAIG. These methods provide useful baselines, but their performance indicates the difficulty of DR generalization under dataset shift. ERM achieves modest AUC values of 66.4 on APTOS, 69.6 on IDRiD, and 70.6 on Messidor, while its F1-scores remain close to 30–34%. This suggests that directly minimizing source-domain classification loss is insufficient to learn representations that transfer robustly across fundus datasets. Similarly, Mixup and MixStyle do not consistently improve performance. Although these approaches can regularize decision boundaries or perturb feature statistics, they are not specifically designed to preserve small lesion evidence or ordinal severity structure, both of which are central to DR grading.

A second line of work incorporates more task-oriented domain generalization mechanisms. Methods such as DRGen, ATS, MDLT, and GDRNet attempt to reduce domain-specific overfitting through data generation, adversarial training, multi-domain learning, or disease-aware representation learning. Among these, GDRNet shows more consistent improvements, achieving AUC values of 69.8 on APTOS, 72.9 on IDRiD, and 78.1 on Messidor, with F1-scores above 35% on all three datasets. These results indicate that DR-specific design choices can improve cross-domain transfer compared with generic regularization. Nevertheless, the absolute F1-scores remain limited, highlighting the persistent difficulty of maintaining class-balanced performance across domains, especially for minority severity grades.

More recent methods have shifted toward attention-based, foundation, and vision-language representations. MIL-ViT and GAD exploit patch-level or multiple-instance reasoning, allowing the model to aggregate evidence from local retinal regions rather than relying only on global image-level features. This is particularly relevant for DR because diagnostic lesions are often sparse, small, and unevenly distributed across the retina. MIL-ViT improves AUC to 80.4 on APTOS, 85.3 on IDRiD, and 81.9 on Messidor, while GAD further improves performance with AUC values of 81.7, 85.5, and 84.0, respectively. CLIP-DR introduces vision-language pretraining to align fundus image features with semantic disease concepts, reaching AUC values above 83 on APTOS and IDRiD and F1-scores above 46% on APTOS and Messidor. These results suggest that richer pretraining and attention-based patch aggregation can improve generalization by learning more transferable disease  representations.

The strongest results in Table 2 are obtained by Dino2-DR, which leverages self-supervised foundation features. Dino2-DR achieves AUC values of 85.6 on APTOS, 88.33 on IDRiD, and 89.24 on Messidor, with the highest F1-scores on APTOS and IDRiD. This reinforces the value of self-supervised pretraining for retinal imaging, where large unlabeled fundus collections can be used to learn anatomical structure, vessel topology, lesion-like textures, and acquisition-invariant features before supervised fine-tuning. The remaining gap between AUC and F1, however, indicates that even strong foundation representations may still struggle with class imbalance, threshold-sensitive decisions, and fine-grained severity separation.

Overall, the related literature shows a clear progression from generic CNN-based empirical risk minimization toward DR-specific domain generalization, multiple-instance reasoning, vision-language learning, and self-supervised foundation models. However, several limitations remain. First, many methods optimize image-level classification without explicitly modeling the ordinal structure of DR severity. Second, generic domain generalization strategies may suppress domain-specific appearance variation but can also fail to preserve high-frequency lesion cues. Third, high AUC does not necessarily imply reliable class-balanced or referral-sensitive performance, particularly under prevalence shift. These limitations motivate PRISM-DR, which combines sample-conditioned normalization, recurrent multi-scale fusion, frequency-aware lesion preservation, Transformer-based scale reasoning, ordinal regression, and prototype regularization to improve robustness while preserving the ordered structure of DR severity grades.

## 3. Proposed PRISM-DR Model for Diabetic Retinopathy Grading

**Figure 3:**
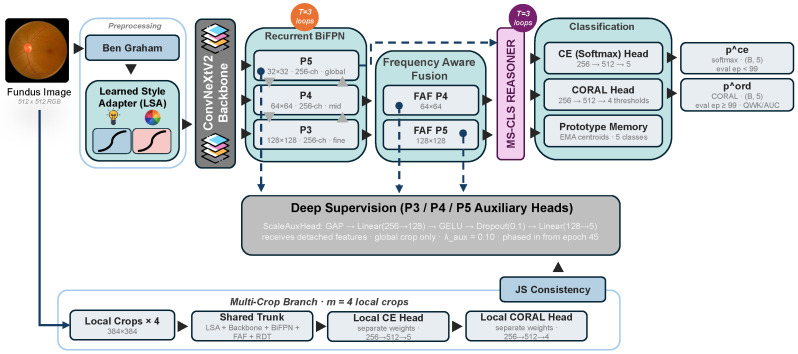
Overview of the PRISM-DR architecture. A fundus image is first optionally processed by the Ben Graham transform and then passed through a Learned Style Adapter (LSA) for sample-conditioned channel modulation. The adapted image is encoded by a ConvNeXtV2-Base backbone, yielding multi-scale feature maps $\left\{C_{3},C_{4},C_{5}\right\}$. A Recurrent BiFPN performs bidirectional iterative fusion with LTI-stable recurrent state updates across T=3 loops, producing refined pyramid levels $\left\{P_{3},P_{4},P_{5}\right\}$. Each of $P_{3}$ and $P_{4}$ is further refined by a Frequency-Aware Fusion (FAF) module that explicitly preserves high-frequency lesion signatures via a learned gating network. A Multi-Scale CLS Reasoner pools each pyramid level into a scale token and processes them with a shared recurrent transformer ($T^{\prime }\;=\;3$ loops, pre-LayerNorm) to produce a single global embedding. The embedding is fed to two parallel output heads with independent pre-projections: a softmax classifier and a CORAL ordinal head. The CORAL pathway is gradient-isolated from the backbone to prevent CE/CORAL gradient conflict. A Prototype Memory Module maintains per-class EMA centroids to regularize the embedding space. Deep supervision is applied via auxiliary heads on each pyramid level. During training the network operates in a multi-crop setting, and a Jensen–Shannon consistency loss encourages agreement between global and local crop predictions.

We propose PRISM-DR (Prototype-Regularized, Instance-Selective Multi-crop Diabetic Retinopathy Grading), a multi-objective framework for five-grade DR severity. The architecture is organized as a feedforward pipeline: a data-driven preprocessing stage followed by a ConvNeXtV2-Base backbone, a Recurrent BiFPN neck for multi-scale feature fusion, a Frequency-Aware Fusion module, a lightweight multi-scale reasoning transformer, dual classification heads with gradient-isolated pathways (categorical and ordinal), and a prototype memory module for embedding regularization. The model is trained in a multi-crop setting with a phased loss curriculum designed for severely imbalanced DR datasets. An overview is shown in Figure 3.

Formally, for an input fundus image I, we generate a set of crops $C=\{I^{\left(g\right)},I^{\left(l_{1}\right)},\ldots ,I^{\left(l_{m}\right)}\}$. Each crop shares the same feature extraction trunk $f_{\theta }\left(\cdot \right)$:

(1) $$e^{\left(c\right)}=f_{\theta }\;\left(I^{\left(c\right)}\right),\;c\in \left\{g,l_{1},\ldots ,l_{m}\right\}.$$

The global crop embedding drives the primary classification; local crop embeddings (m=4) provide auxiliary supervision and multi-view consistency regularization.

### 3.1. Preprocessing Pipeline

**Figure 4:**
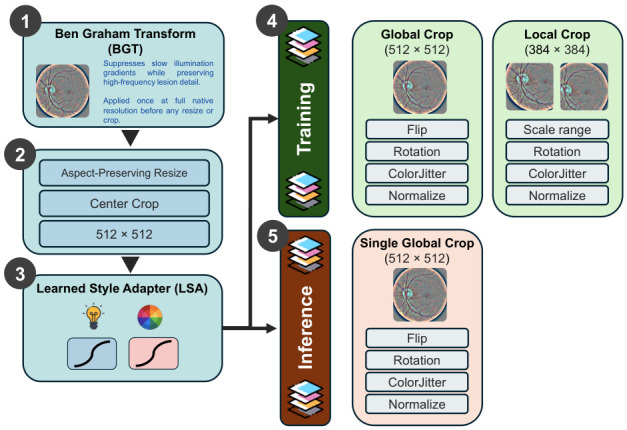
PRISM-DR preprocessing pipeline. Stages 1–2 are applied offline at native image resolution and are deterministic (no learned parameters). Stage 3 (Learned Style Adapter) is in-model and trained jointly with the backbone. Stage 4 and Stage 5 diverges by mode: training produces one global crop (512×512) and four independently augmented local crops (384×384); inference produces a single global crop with no augmentation. All crops share the same backbone $f_{\theta }$ and are ImageNet-normalized before entering the feature extraction trunk.

The preprocessing pipeline transforms raw fundus images of arbitrary resolution and device origin into a normalized, model-ready crop batch. It is organized as four sequential stages: two offline, deterministic transformations applied once per image at native resolution; one in-model learnable normalization applied jointly with the backbone; and one mode-dependent crop extraction stage whose behavior differs between training and inference. Figure 4 provides a schematic overview.

**Ben Graham Transform (BGT).** We apply the classical Ben Graham local-average subtraction [51] as defined in Equation (2).

(2) $$\mathrm{BGT}\left(I\right)=4I-4\;G_{\sigma }*I+128,$$

where $G_{\sigma }$ is a Gaussian blur with σ=10. BGT suppresses slow illumination gradients arising from non-uniform flash illumination and lens vignetting while preserving high-frequency vascular and lesion detail. Critically, the transform is applied once at native capture resolution before any resize operation: applying it post-resize would shift the effective spatial frequency cutoff relative to lesion size in a device-dependent manner, since σ is specified in absolute pixels rather than as a fraction of image width.

**Aspect-preserving resize and center crop**. Following BGT, images are resized so that the shorter edge equals 512 px (preserving aspect ratio), and a 512×512 center crop is extracted. This establishes a canonical spatial resolution shared by all devices and all downstream processing steps. From this point onward, all pixel coordinates (including GradCAM maps, lesion bounding-box projections, and FAF gate activations) reference the 512×512 coordinate frame. No padding is introduced; the center crop discards peripheral black borders common in fundus photography without affecting the macula-centered field of view.

**Learned Style Adapter (LSA).** To compensate for residual device-specific color shifts that survive BGT, a lightweight **Learned Style Adapter** performs a sample-conditioned affine modulation of the RGB channels. A two-layer CNN encoder and global average pooling extract a style descriptor, which is decoded into per-channel scale and bias parameters $\left(\gamma ,\beta \right)\in R^{3}$:

(3) $$\mathrm{LSA}\left(I\right)=I⊙\;\left(1+\alpha \tanh(\gamma )\right)+\alpha \beta ,$$

where ⊙ denotes channel-wise multiplication and α=0.1 limits the modulation strength to prevent content distortion. It performs a mild, learned white-balance correction rather than a global color remapping. The LSA is trained with all other parameters using the same learning rate group as the classification heads, and has fewer than 64 K parameters. Notably, the LSA is the *only* learnable component in the preprocessing pipeline; Stages 1 and 2 are entirely fixed and produce deterministic outputs for a given image.

**Multi-crop extraction.** After LSA modulation, training and inference paths diverge.

**Training.** Each image generates five crops processed in a single forward pass through the shared backbone $f_{\theta }$. One global crop (512×512) is obtained by center-cropping the already-resized image; it provides the primary evidence for the classification heads and drives all main losses. Four local crops (384 × 384) are drawn by RandomResizedCrop(384,scale=[0.25,0.65]), sampling distinct partial views of the retina. Both crop branches are independently augmented with random horizontal and vertical flips (p=0.5), full $360^{\circ }$ rotation (fundus images have no canonical orientation), and color jitter (brightness/contrast ±0.15, saturation ±0.10, hue ±0.02 for the global crop; slightly stronger at ±0.18 for local crops). All crops are finally normalized with ImageNet channel statistics (μ=(0.485,0.456,0.406), σ=(0.229,0.224,0.225)).

**Inference.** A single global crop (512×512) is produced by center-cropping with no augmentation. All inference metrics, including validation QWK, cross-dataset generalization, and XAI spatial alignment, are computed on this single deterministic forward pass using the EMA shadow model weights. For GradCAM and FAF gate computation, gradients are re-enabled for the target layers while all other pipeline stages remain in evaluation mode.

### 3.2. ConvNeXtV2-Base Backbone

We employ a ConvNeXtV2-Base backbone [14] in features-only mode with out_indices  = (1, 2, 3), yielding intermediate feature maps $\left\{C_{3},C_{4},C_{5}\right\}$ at progressively coarser resolutions. Higher-resolution maps preserve microaneurysms and hemorrhage boundaries; deeper maps capture global proliferative patterns. The backbone is initialized from ImageNet-1k pretrained weights and trained with a layer-wise learning rate decay (LLRD) factor of 0.1× relative to the head learning rate to protect low-level representations.

### 3.3. Recurrent BiFPN

We employ a Recurrent BiFPN that performs T=3 bidirectional fusion loops over the feature pyramid, sharing all convolution weights across loops. This trades parameter count for iterative refinement depth. The word “recurrent” in our naming refers exclusively to depth-wise iterative refinement, the repeated application of the same parameterized operation to the same spatial representation until convergence. This is a well-established usage in the non-temporal deep learning literature (deep equilibrium models [52,53], unrolled optimization [54], iterative BiFPN [55]).

**Bidirectional fusion**. In each loop *t*, a top-down pass fuses global context from $P_{5}$ into $P_{3}$, and a subsequent bottom-up pass propagates fine-grained lesion cues back upward via max-pooling for downsampling. For a top-down update of $P_{4}$:

(4) $$P_{4}^{(t),\mathrm{td}}=\mathrm{Refine}\;\left(\frac{w_{1}P_{4}+w_{2}\;\mathrm{Up}\left(P_{5}^{(t),\mathrm{td}}\right)}{w_{1}+w_{2}+\epsilon }\right),$$

where $w_{1},w_{2}\geq 0$ are per-level learnable fast-normalized weights (ReLU-constrained) and Up(·) is bilinear upsampling. The refinement block is a shared 3 × 3 Conv → BatchNorm → GELU → Dropout(p=0.20) sequence.

**LTI-stable recurrent states.** Each pyramid level k∈{3,4,5} maintains a hidden state $x_{k}^{\left(t\right)}\in R^{d_{f}\times h_{k}\times w_{k}}$ across loops. The transition is parameterized as a Linear Time-Invariant (LTI) system to guarantee stability:

(5) $$\begin{array}{cc} A_{k} & =\exp\;\left(-\exp(\left(\log\Delta_{k}+\log A_{k}^{0}\right)\mathrm{clamp}\left(-20,20\right))\right)\in \left(0,1\right), \end{array}$$

(6) $$\begin{array}{cc} x_{k}^{\left(t+1\right)} & =A_{k}⊙x_{k}^{\left(t\right)}+\left|B_{k}\right|⊙x_{k}^{\left(0\right)}+\mathrm{Refine}\left(x_{k}^{\left(t\right)}\right), \end{array}$$

where $A_{k}$ is a learned decay parameter clamped in (0,1) via log-space exponentiation (initialized at $\log A_{k}^{0}\;=\;-2.0$, giving $A_{k}\approx 0.87$) and $x_{k}^{\left(0\right)}$ is the initial projection of $C_{k}$. Re-injecting the initial feature every loop prevents gradient drift in deep iterative refinement. The re-injection weight $B_{k}$ is initialized at 0.1 and learned jointly. Per-loop GroupNorm is applied after each state update to stabilize accumulation.

**Loop embeddings.** A sinusoidal loop embedding indexed by *t* is injected additively into each pyramid level before refinement, making the shared convolution weights loop-index-aware without additional parameters.

All levels are projected to a common channel dimension $d_{f}\;=\;256$ and the neck output is $\left\{P_{3},P_{4},P_{5}\right\}$.

### 3.4. Frequency-Aware Fusion (FAF)

DR lesions (microaneurysms, exudate edges, hemorrhage boundaries) manifest as high-frequency structures. We apply a Frequency-Aware Fusion module to $P_{3}$ and $P_{4}$ independently. The input feature map Z is decomposed into low- and high-frequency components, as presented in Equation (7).

(7) $$\begin{array}{cc} Z_{\mathrm{low}} & ={\mathrm{AvgPool}}_{3\times 3}\left(Z\right),\;Z_{\mathrm{high}}=Z-Z_{\mathrm{low}}. \end{array}$$

A two-stage gating network produces a spatially adaptive retention weight:

(8) $$\begin{array}{cc} h & =\mathrm{GELU}\;\left({\mathrm{Conv}}_{1\times 1}^{2d_{f}\to d_{f}}\left(\left[Z_{\mathrm{low}},Z_{\mathrm{high}}\right]\right)\right), \end{array}$$

(9) $$\begin{array}{cc} g & =\sigma \;\left({\mathrm{Conv}}_{1\times 1}^{d_{f}\to 1}\left(\mathrm{Dropout}\left(h\right)\right)\right), \end{array}$$

(10) $$\begin{array}{cc} \hat{g} & =0.1+0.9\;g,\;\mathrm{FAF}\left(Z\right)=Z_{\mathrm{low}}+\hat{g}⊙Z_{\mathrm{high}}. \end{array}$$

The intermediate projection contracts the concatenated $2d_{f}$-channel input to $d_{f}$ with a nonlinear activation before the scalar gating convolution, providing capacity to model channel interactions before spatial gating. The gate $\hat{g}$ is clamped to retain at least 10% of high-frequency content regardless of the learned gating value, preventing catastrophic suppression of fine lesion details.

**Spatial resolution and fine-lesion retention.** Resizing fundus images (e.g., 512×512) introduces a trade-off between computational tractability and the preservation of fine-scale pathological structures. Individual microaneurysms, whose diameter at the retinal plane typically corresponds to fewer than 3 pixels at this resolution, cannot be spatially resolved after downsampling. Three design decisions partially mitigate this constraint. The BGT (Equation (2)) is applied at full native resolution before any resize, amplifying high-frequency lesion content in the native coordinate frame where it is most spatially distinct. The FAF module subsequently recovers residual high-frequency energy at the feature level through its learned spatial gate, operating at $P_{3}$ feature resolution (128×128) rather than relying on global pooling. The multi-crop training strategy exposes the model to retinal subregions at higher effective magnification through four local crops sampled at scale [0.25,0.65] of the 512×512 image. Empirically, the model achieves a Pointing Game accuracy for microaneurysms that is 10.3× above the random baseline and an Energy Ratio Enrichment of 3.67×, indicating that the model learns to attend to the vascular neighborhood context associated with MA clustering even when individual lesion instances are below the resolution limit. Whether higher input resolutions would improve fine-lesion localization remains an open question for future investigation.

### 3.5. Multi-Scale CLS Reasoner (Recurrent Depth Transformer)

To aggregate evidence across spatial scales, we introduce a Multi-Scale CLS Reasoner that processes scale tokens with a recurrent depth transformer (RDT). Global-average-pooling is applied to each pyramid level to yield three scale tokens:

(11) $$s_{k}=\mathrm{GAP}\left(P_{k}\right)\in R^{d_{f}},\;k\in \left\{3,4,5\right\}.$$

A learnable [CLS] token $c^{\left(0\right)}\in R^{d_{f}}$ is prepended, and learnable scale position embeddings are added to each $s_{k}$.

A single TransformerEncoderLayer (8 heads, $d_{\mathrm{ff}}\;=\;1024$, pre-LayerNorm, Dropout (*p* = 0.10)) is applied $T^{\prime }\;=\;3$ times with shared weights. Pre-LayerNorm (norm_first=True) is essential for numerical stability in the recurrent setting. Before each iteration *t*, a sinusoidal loop embedding indexed by *t* is added to the CLS token:

(12) $$\left[c^{\left(t+1\right)},s_{3}^{\left(t+1\right)},s_{4}^{\left(t+1\right)},s_{5}^{\left(t+1\right)}\right]=\mathrm{TF}\;\left([c^{\left(t\right)}+\mathrm{LE}\left(t\right),\;s_{3}^{\left(t\right)},s_{4}^{\left(t\right)},s_{5}^{\left(t\right)}]\right).$$

The final normalized CLS token $e=\mathrm{LN}\left(c^{\left(T^{\prime }\right)}\right)\in R^{256}$ serves as the global embedding for classification. Sharing weights across iterations implements recurrent depth: computational depth grows without proportional parameter growth, enabling efficient cross-scale reasoning.

### 3.6. Dual Classification Heads with Gradient Isolation

The global embedding e is fed to two parallel output heads. Each head maintains an independent pre-projection layer to prevent CE and CORAL gradients from interfering through a shared feature transformation, as tied projections cause gradient conflict between the categorical and ordinal objectives.

**Softmax (CE) head** is defined in Equation (13) with $W_{1}^{\mathrm{ce}}\in R^{512\times 256}$ and $W_{2}^{\mathrm{ce}}\in R^{5\times 512}$, each followed by Dropout (0.10).

(13) $$z^{\mathrm{ce}}=W_{2}^{\mathrm{ce}}\;\mathrm{GELU}\left(W_{1}^{\mathrm{ce}}e\right),\;p^{\mathrm{ce}}=\mathrm{softmax}\left(z^{\mathrm{ce}}\right),$$

**CORAL ordinal head.** To exploit the ordinal structure of DR grades (0–4), we use a CORAL head with its own independent pre-projection $W_{1}^{\mathrm{cor}}\in R^{512\times 256}$:

(14) $$o=W_{\mathrm{cor}}\;\mathrm{GELU}\left(W_{1}^{\mathrm{cor}}e\right)+b,\;p_{k}=\sigma \left(o_{k}\right)\approx P\left(y>k\right),$$

where $W_{\mathrm{cor}}\in R^{1\times 512}$ is a shared weight vector and $b\in R^{K-1}$ are per-threshold biases initialized from training-set class frequency quantiles. Biases b are sorted in descending order at each forward pass to enforce the monotonicity constraint P(y>0)≥P(y>1)≥…. Class probabilities are recovered as

(15) P(y=c)=P(y>c−1)−P(y>c),

with boundary terms P(y=0)=1−P(y>0) and P(y=K−1)=P(y>K−2), normalized to sum to one.

**Gradient isolation and design rationale.** The CORAL pathway receives gradients from the ordinal loss only: the backbone sees no gradient from the CORAL objective (coral_detach_backbone=True). This is a permanent architectural property of PRISM-DR’s training procedure, not a transient warm-up strategy.

The decision arose from empirical evaluation of the full-backpropagation configuration. When CORAL gradients reached the backbone at the initial weight $\lambda_{\mathrm{coral}}=1.0$, the composite gradient norm (raw_grad_norm) consistently exceeded 17, causing training instability and degraded validation QWK. Reducing $\lambda_{\mathrm{coral}}$ to 0.3 with cosine annealing and applying global gradient clipping at norm 1.0 partially stabilized the norms but did not recover the QWK degradation. The root cause is a curvature mismatch: softmax cross-entropy and per-threshold binary cross-entropy (CORAL) operate on different output manifolds and generate structurally conflicting gradient directions in the shared backbone parameters. Gradient clipping dampens but does not resolve this conflict.

The detachment assigns the backbone exclusively to the CE, prototype, and consistency objective cluster, while the CORAL head specializes its own projection. Ordinal inductive bias reaches the backbone through two indirect pathways: (i) severity-weighted class penalties in the CE loss, whose weights increase monotonically with ICDR grade; and (ii) the prototype contrastive loss, which enforces grade-indexed cluster separation in the embedding space that the CORAL threshold geometry relies on.

**Deep supervision.** Auxiliary softmax heads are attached to $P_{3}$, $P_{4}$, and $P_{5}$, respectively, and supervised with cross-entropy on the global crop only. Gradients are detached from the BiFPN to prevent destabilization.

### 3.7. Prototype Memory Module

To encourage class-consistent embeddings and improve inter-class separability, we maintain a Prototype Memory Module (PMM) consisting of EMA centroids ${\left\{\mu_{c}\right\}}_{c=0}^{K-1}$ in the normalized embedding space. After each optimization step, centroids are updated from all available crops (global and local) to avoid global-crop bias:

(16) $$\mu_{c}\leftarrow \beta_{t}\;\mu_{c}+\left(1-\beta_{t}\right)\;{\bar{e}}_{c},\;{\bar{e}}_{c}=\frac{1}{|B_{c}|}\sum_{i\in B_{c}}\frac{e_{i}}{∥e_{i}{∥}_{2}},$$

where $\beta_{t}$ is linearly scheduled from 0.99 at epoch 0 to 0.999 at the final epoch to allow rapid early adaptation before stabilizing [56]. In multi-GPU training, prototype banks are synchronized across ranks via all-reduce averaging after each update. Prototype logits are computed via temperature-scaled cosine similarity:

(17) $$z_{c}^{\mathrm{proto}}=\frac{1}{\exp(\tau )}\frac{e^{⊤}\mu_{c}}{{∥e∥}_{2}\;{∥\mu_{c}∥}_{2}},$$

where τ is a learnable log-temperature parameter (initialized at log0.07) constrained to be positive. A cross-entropy prototype loss $L_{\mathrm{Proto}}$ regularizes the embedding space. The value τ = 0.07 is the canonical initialization for temperature-scaled cosine similarity in contrastive and prototype learning. It was introduced as the empirically optimal temperature in SimCLR [57] for normalized embedding spaces.

### 3.8. Multi-Crop Training and Phased Loss Curriculum

**Multi-crop setting.** The trunk is shared across all crops. A global crop of 512×512 and m=4 local crops of 384×384 (sampled with scale ∈[0.25,0.65]) are processed in each forward pass. Separate CE and CORAL heads are instantiated for local crops to encourage complementary evidence.

**Objective function.** The full training loss is defined in Equation (18).

(18) $$L=\lambda_{\mathrm{ce}}L_{\mathrm{CE}}+\lambda_{\mathrm{coral}}L_{\mathrm{CORAL}}+\lambda_{\mathrm{proto}}L_{\mathrm{Proto}}+\lambda_{\mathrm{aux}}L_{\mathrm{Aux}}+\lambda_{\mathrm{cons}}L_{\mathrm{Cons}}.$$

**Cross-Entropy** $L_{\mathrm{CE}}$: label-smoothed (ϵ=0.05) cross-entropy with per-class weights proportional to the square-root inverse of class frequency to mitigate the heavy No-DR (y=0) majority. When used in conjunction with the class-balanced distributed sampler, weights are halved to avoid double-amplification.

**CORAL loss** $L_{\mathrm{CORAL}}$: binary cross-entropy summed over the K−1 ordinal thresholds:

(19) $$L_{\mathrm{CORAL}}=\frac{1}{K-1}\sum_{k=0}^{K-2}\mathrm{BCE}\;\left(\sigma \left(o_{k}\right),\;1\left[y>k\right]\right).$$

**Prototype loss** $L_{\mathrm{Proto}}$: cross-entropy over prototype logits (Section 3.7).

**Scale auxiliary loss** $L_{\mathrm{Aux}}$: mean of cross-entropy losses from the three deep-supervision heads on the global crop; gradients are detached from the BiFPN to prevent destabilization.

**View-consistency loss:** $L_{\mathrm{Cons}}$: Jensen–Shannon divergence between the global and each local crop’s softmax distribution:
  (20) $$L_{\mathrm{Cons}}=\frac{1}{m}\sum_{j=1}^{m}\mathrm{JS}\;\left(p^{\left(g\right)}\;∥\;p^{\left(l_{j}\right)}\right).$$

**Phased curriculum.** To prevent early collapse to the majority class on imbalanced DR data, we employ a four-phase loss schedule. All phases are parameterized as fractions of total training epochs (E=300):

1. *Warm-up* (epochs 0–20): $L_{\mathrm{CE}}$ only ($\lambda_{\mathrm{ce}}\;=\;0.5$). Allows backbone and neck to stabilize before multi-head training.
2. *CORAL ramp* (epochs 20–45): $\lambda_{\mathrm{coral}}$ cosine-annealed from 0.01 to 0.3. The CORAL head is gradient-isolated from the backbone (coral_detach_backbone=True); this isolation persists for all subsequent phases and is a permanent property of the training procedure.
3. *Auxiliary ramp* (epochs 45–99): $\lambda_{\mathrm{proto}}$, $\lambda_{\mathrm{aux}}$, $\lambda_{\mathrm{cons}}$ cosine-annealed from 0 to their target values. The wider ramp window (54 epochs vs. the previous 30-epoch window) was adopted empirically to prevent validation loss spikes observed when auxiliary losses activate too sharply.
4. *Full training* (epoch ≥ 99): all losses active at final weights ($\lambda_{\mathrm{ce}}\;=\;0.5$, $\lambda_{\mathrm{coral}}\;=\;0.3$, $\lambda_{\mathrm{proto}}\;=\;0.15$, $\lambda_{\mathrm{aux}}\;=\;0.10$, $\lambda_{\mathrm{cons}}\;=\;0.05$).

### 3.9. Interpretability via Multi-Scale GradCAM

Reliable deployment of automated DR grading requires not only accurate predictions but also evidence that the model attends to clinically plausible regions. PRISM-DR provides built-in multi-scale interpretability through class-discriminative activation maps derived from six spatially informative internal layers, without requiring pixel-level lesion annotations or post-hoc surrogate models.

**Targeted layers.** Forward hooks and retained gradients are registered on the following layers (Table 3):

**Computation.** For a target class *c*, the gradient of the CE logit $z_{c}^{\mathrm{ce}}$ with respect to the activation tensor $A^{\left(l\right)}\in R^{d\times h_{l}\times w_{l}}$ of layer *l* is computed via a single backward pass. Channel importance weights are obtained by global average pooling over spatial dimensions:

(21) $$\alpha_{k}^{\left(l\right)}=\frac{1}{h_{l}w_{l}}\sum_{i,j}\frac{\partial z_{c}^{\mathrm{ce}}}{\partial A_{k,i,j}^{\left(l\right)}},$$

and the class-discriminative activation map is

(22) $${\mathrm{CAM}}^{\left(l\right)}=\mathrm{ReLU}\;\left(\sum_{k}\alpha_{k}^{\left(l\right)}A_{k}^{\left(l\right)}\right),$$

bilinearly upsampled to the input resolution 512×512 and min-max normalized to [0,1]. FAF gate maps are captured directly from the forward pass (no gradient required) as they encode the spatially varying high-frequency retention weights $\hat{g}\in \left[0.1,1.0\right]$.

## 4. Experiments

This section describes the experimental protocol used to evaluate our proposed method. We first define the evaluation scenarios considered in this study, covering both in-domain validation and cross-dataset generalization. We then summarize the baseline models used for comparison, including general-purpose computer-vision backbones and retina-specific foundation models. Next, we describe the datasets, followed by the evaluation metrics adopted to quantify agreement with reference grades. Finally, we report the experimental setup, including the implementation details, training hyperparameters, and the computing environment.

### 4.1. Evaluation Scenarios

We evaluate PRISM-DR under three complementary scenarios. The first two are quantitative and address distribution shift at increasing severity: in-domain evaluation (no domain shift) and the Fixed-Source, Multi-Target (FSMT) protocol, inspired by the fixed-domain benchmarking strategy of [28]. The third scenario is qualitative and evaluates whether the model’s internal attention patterns generalize across domains in a clinically meaningful way. In the classical In-domain evaluation (ID), training and evaluation are performed within the same dataset. In FSMT, the training domains are fixed *a priori*, and model performance is assessed by transferring to multiple unseen target datasets without any target-domain adaptation. This design reduces the number of training runs compared with classic domain generalization (DG) benchmarking while still directly probing robustness to acquisition and cohort shift.

**FSMT-1 (single-source, multi-target).** In FSMT-1, we fix *one* dataset as the sole training domain. The model is trained and tuned using only this source dataset, with an internal split where 20% of the source data is reserved for validation and the remaining 80% used for training. The final model is then evaluated on *each* of the remaining datasets as independent target domains. This setting corresponds to an *extreme single-domain generalization* regime, closely related to the ESDG test setting described in [38], and is particularly challenging when the chosen source domain is narrow in device diversity or class distribution.

**FSMT-2 (two-source, multi-target).** In FSMT-2, we fix two training domains in advance, selecting the two largest and most diverse cohorts: EyePACS and DDR. The model is trained on the union of these datasets, with 20% of their combined samples held out for validation and the remaining 80% used for training. All other datasets are treated as external targets and used exclusively for cross-domain evaluation (multi-target validation), providing a pragmatic robustness test that better matches real deployment where training data often comes from a limited number of large screening programs.

We contrast FSMT with the leave-one-domain-out DG test protocol popularized in GDRBench [38,58], where each domain is held out in turn for evaluation and the model is trained on the remaining domains. While informative, leave-one-domain-out requires multiple full trainings (one per held-out domain), making it computationally intensive; additionally, it does not isolate *single-source* generalization in the same way as FSMT-1. By fixing training domains, FSMT yields a controlled, reproducible evaluation of transfer to multiple targets under a fixed training budget, while still capturing meaningful cross-dataset shift.

**Qualitative interpretability evaluation.** Beyond aggregate performance scores, we assess model generalization qualitatively through class-discriminative activation maps obtained via GradCAM [59]. This scenario tests whether the spatial attention learned from grade-level supervision is clinically plausible (i.e., aligned with known DR lesion locations), and probes whether those attention patterns remain coherent under domain shift when the model is applied to unseen target datasets. Interpretability evaluation is performed separately on each test dataset and organized as per-grade facegrids that allow direct visual comparison of grade-specific attention across Grades 0–4.

**Quantitative interpretability evaluation.** To address the limitation of purely qualitative assessment, we additionally measure the statistical alignment between the model’s activation maps and expert-annotated lesion bounding boxes from the DDR lesion-detection split [60]. This evaluation covers 755 annotated images with bounding boxes for four lesion types: microaneurysms (MA), hemorrhages (HE), hard exudates (EX), and soft exudates (SE). No lesion annotations are used at any stage of training; the alignment metrics are applied strictly at evaluation time to probe whether the representations learned under grade-level supervision encode lesion-relevant spatial structure. The full metric definitions, implementation details, and coordinate-projection pipeline are described in Section 4.4.2, and results are reported in Section 5.4.

### 4.2. Baseline Models

We compare our model against a diverse set of baseline approaches, including retinal foundation models, large vision–language models, and representative state-of-the-art methods reported in recent literature.

#### 4.2.1. RETFound and RETFound-Green

RETFound [20] and RETFound-Green [34] are retinal foundation models pretrained using masked autoencoding (MAE) on large-scale fundus image collections. These models provide pretrained vision backbones but do not include task-specific classification heads for DR grading. RETFound did its self-supervised pretraining on approximately 904k CFP and 736k OCTs; for CFP, about 90% came from MEH-MIDAS and approximately 10% from EyePACS dataset. RETFound-Green was pretrained on 75k publicly available retinal images (a strict subset of DERETFound’s public data). The authors enumerate public datasets used in the manuscript such as DDR, IDRiD, Messidor-2, ODIR-2019, BRSET, AIROGS and ROP.

Following standard evaluation practices for foundation models without finetuning, we adopt a *k*-nearest neighbors (KNN) protocol on extracted image features. Specifically, each retinal image is forwarded through the pretrained MAE ViT-Large backbone, and a fixed-length 1024-dimensional feature vector is obtained from the final representation layer. These features are then classified using a distance-weighted KNN classifier with k=5. To reduce bias and avoid optimistic estimates, we employ 5-fold cross-validation, where predictions for each sample are obtained from a KNN model fitted on the remaining folds. This protocol evaluates the intrinsic quality and separability of the learned representations without introducing additional trainable parameters.

#### 4.2.2. MedGemma-4B

MedGemma-4B [10] is a large-scale multimodal foundation model designed for medical applications. MedGemma does include ophthalmology/fundus data in its training mix and EyePACS dataset is explicitly named. In this study, we utilize its visual encoding and classification capabilities to perform DR severity grading according to the ICDR five-grade scale.

Different from RETFound-based baselines, MedGemma-4B is evaluated using its classification output directly, mapping predictions to the corresponding DR severity classes. This setup reflects a realistic usage scenario in which the model is applied as a pretrained medical vision–language system, without architectural modification.

### 4.3. Datasets

We utilize six publicly available datasets for our experiments. We present the summarization in Table 4.

- **EyePACS** [61] contains over 35,000 retinal images with DR severity labels (0–4). It is a large-scale DR screening dataset released through the Kaggle Diabetic Retinopathy Detection challenge. It contains high-resolution CFP acquired under highly variable real-world conditions (camera models, illumination, focus, and field-of-view), which makes it a common choice for training models intended to generalize across acquisition settings.
- **APTOS 2019** [62] Blindness Detection dataset was released for a Kaggle competition focused on automated DR severity assessment. It provides 3662 labeled fundus images with 5-class DR grades (0–4) and includes an additional unlabeled test split in the original competition setting.
- **IDRiD** [63] (Indian Diabetic Retinopathy Image Dataset) is a curated benchmark designed to support both image-level disease grading and lesion-centric evaluation in diabetic retinopathy. The dataset comprises 516 high-resolution fundus images (commonly reported at 4288 × 2848) with expert-provided DR severity grades and DME grades; the dataset documentation also specifies a standard grading split (e.g., 413/103 images) used in many studies.
- **mBRSET** [64] (Mobile Brazilian Retinal Dataset) is a portable-camera retinal dataset aimed at enabling research under conditions typical of low-resource and field screening contexts. The PhysioNet release reports 5164 images from 1291 patients, and the dataset is accompanied by clinical/demographic metadata to support analyses beyond pure image classification (e.g., bias, subgroup performance, and multimodal prediction). Images were captured using a handheld, smartphone-based fundus camera system (Phelcom Eyer), which introduces acquisition characteristics distinct from tabletop clinical devices and is therefore valuable for domain-shift evaluation.
- **Messidor-2** [65] is a well-known DR screening benchmark organized as examinations, each containing two macula-centered fundus images (one per eye). It includes 874 examinations (1748 images) and is frequently used for evaluating referable DR detection and generalization.
- **DDR** [60] (Diabetic Retinopathy Dataset) is a large, multi-center dataset developed to support DR algorithm development at scale. Public descriptions report 13,673 color fundus images collected from 147 hospitals across China, with DR severity labeled according to a 5-stage scheme and additional assessment of image gradability. Additionaly, the DDR dataset provides high-quality, pixel-level and bounding-box annotations for 755 images, identifying four specific lesion types: microaneurysms (MA), hemorrhages (HE), hard exudates (EX), and soft exudates (SE).

### 4.4. Evaluation Metrics

DR grading is an imbalanced, ordinal, multi-class problem (ICDR grades 0–4). To obtain a comprehensive view of model behavior, we report several complementary metrics, each highlighting a different aspect of performance.

- **Quadratic Weighted Kappa (QWK)** is the primary metric used in DR grading benchmarks and competitions. It measures the agreement between the model’s predictions and the ground truth on an ordinal scale, penalizing large disagreements more heavily than small ones. It is defined in Equation (23).
  (23) $$\kappa =1-\frac{\sum_{i,j}w_{i,j}O_{i,j}}{\sum_{i,j}w_{i,j}E_{i,j}},$$
  where $O_{i,j}$ is the observed confusion matrix, $E_{i,j}$ is the expected confusion matrix under random agreement, and $w_{i,j}$ is the quadratic weight matrix. QWK explicitly exploits the ordinal nature of DR grades, making it well aligned with clinical severity (e.g., misclassifying 0 as 1 is less severe than 0 as 4). However, it does not directly separate performance per class and can be less intuitive to interpret than threshold-based metrics.
- Overall **Accuracy** is defined in Equation (24) as the proportion of correctly classified samples.
  (24) $$\mathrm{Acc}=\frac{1}{N}\sum_{n=1}^{N}1\left({\hat{y}}_{n}=y_{n}\right),$$
  where *N* is the number of samples, $y_{n}$ is the true label, and ${\hat{y}}_{n}$ is the predicted label. Accuracy is simple and intuitive, but in highly imbalanced DR datasets (e.g., many no-DR images), it can be dominated by the majority classes and thus overestimate clinical usefulness.
- **Macro F1-Score**: Let the precision and recall for class *k* be defined as in Equation (25) and the corresponding F1-score. The macro F1-score is then obtained by averaging $F1_{k}$ over all classes according to Equation (26).
  (25) $${\mathrm{Prec}}_{k}=\frac{TP_{k}}{TP_{k}+FP_{k}},\;{\mathrm{Rec}}_{k}=\frac{TP_{k}}{TP_{k}+FN_{k}},$$
  (26) $$F1_{k}=2\cdot \frac{{\mathrm{Prec}}_{k}\cdot {\mathrm{Rec}}_{k}}{{\mathrm{Prec}}_{k}+{\mathrm{Rec}}_{k}},\;F1_{\mathrm{macro}}=\frac{1}{K}\sum_{k=1}^{K}F1_{k}$$
  where *K* is the number of classes. Macro F1 treats all DR grades equally and is informative under class imbalance (e.g., few proliferative DR cases). Its main limitation is that it ignores the ordinal relationships between grades and depends on a fixed decision threshold.
- **Macro ROC-AUC (AUC)**: For probabilistic outputs, we compute the Area Under the Receiver Operating Characteristic curve (ROC-AUC) in a one-vs-rest fashion for each class and then average, as demonstrated in Equation (27).
  (27) $${\mathrm{AUC}}_{\mathrm{macro}}=\frac{1}{K}\sum_{k=1}^{K}{\mathrm{AUC}}_{\mathrm{ROC}}^{\left(k\right)}.$$
  ROC-AUC measures the model’s ability to rank images from negative to positive for each class across all possible thresholds. It is threshold-independent and relatively robust to class imbalance, providing a good notion of ranking quality (e.g., how well high-severity DR cases are pushed to the top). However, ROC-AUC may still appear optimistic when the positive class is extremely rare.
- **Macro PR-AUC (AUCPR)**: We also report the Area Under the Precision–Recall curve (PR-AUC) in a one-vs-rest setting and average across classes, as defined in Equation (28).
  (28) $$\begin{array}{c} {\mathrm{AUC}}_{\mathrm{PR}}^{\left(k\right)}=\int_{0}^{1}{\mathrm{Prec}}_{k}\left({\mathrm{Rec}}_{k}\right)\;d{\mathrm{Rec}}_{k},\;\\ {\mathrm{AUC}}_{\mathrm{PR},\;\mathrm{macro}}=\frac{1}{K}\sum_{k=1}^{K}{\mathrm{AUC}}_{\mathrm{PR}}^{\left(k\right)}, \end{array}$$
  AUCPR focuses on the performance for the positive class at varying thresholds and is more sensitive to class imbalance than ROC-AUC. In DR, it is more informative for higher-severity grades, which are rare but clinically critical. The downside is that AUCPR can be more variable and less intuitive to interpret numerically than ROC-AUC.

We chose metrics that provide complementary perspectives on model behavior in DR grading. QWK captures agreement on the ordinal severity scale and is our primary ranking metric. Accuracy provides an overall view but can hide failures on minority, high-risk grades. Macro F1 and AUCPR emphasize performance on underrepresented classes and error trade-offs relevant for screening and referral decisions. The macro ROC-AUC summarizes the quality of the predicted risk ranking independently of any specific operating threshold. By analyzing all of them jointly, we obtain a more reliable and clinically meaningful assessment than any single metric alone.

#### 4.4.1. Interpretability Evaluation Protocol

This subsection details the technical implementation of the qualitative interpretability scenario introduced in Section 4.1. Interpretability evaluation is conducted by extracting class-discriminative activation maps using Gradient-weighted Class Activation Mapping (GradCAM) [59] from six spatially informative internal layers of PRISM-DR. The goal is to verify that the model attends to anatomically plausible regions for each DR severity grade, and that these attention patterns transfer coherently to unseen target datasets, without using any pixel-level lesion annotations during training or evaluation.

**Targeted layers**. Hooks are registered on six internal layers that collectively span the model’s spatial hierarchy: (i) FAF output at $P_{3}$ (fine scale, 64×64 feature resolution), capturing microaneurysm-scale cues; (ii) FAF output at $P_{4}$ (mid scale), capturing hemorrhage and capillary dropout patterns; (iii) $P_{5}$ input to the MS-CLS Reasoner (global scale), encoding overall lesion burden and optic disc context; (iv) the deepest ConvNeXtV2 backbone stage $C_{5}$, reflecting high-level semantic features; and (v, vi) the learned FAF gate maps $\hat{g}$ at $P_{3}$ and $P_{4}$, which are captured without gradient computation and reveal the spatial distribution of high-frequency content retention.

**Gradient computation.** For each test image, a forward pass computes the CE logit $z_{c}^{\mathrm{ce}}$ for the target class *c* (set to the predicted grade by default). A single backward pass retains gradients at each hooked layer. Channel importance weights $\alpha_{k}^{\left(l\right)}$ are computed by global average pooling over spatial positions, and the class activation map is obtained as ${\mathrm{CAM}}^{\left(l\right)}=\mathrm{ReLU}\left(\sum_{k}\alpha_{k}^{\left(l\right)}A_{k}^{\left(l\right)}\right)$, bilinearly upsampled to 512×512 and min-max normalized to [0,1].

**Per-grade facegrid.** Qualitative evaluation is organized as per-grade facegrids. For each ICDR grade (0–4), four representative images are selected from the evaluation set and assembled into a 4×10 facegrid: each grade occupies two columns (original fundus image and GradCAM overlay from $P_{3}$), ordered by grade from No DR to Proliferative DR. Facegrids are generated separately for each evaluation dataset to assess whether attention patterns are consistent across domain shifts. Overlay heatmaps use the jet colormap with α=0.5 blending.

#### 4.4.2. Quantitative Interpretability Evaluation Protocol

This subsection defines the metrics, annotation source, and coordinate-projection pipeline used to evaluate the spatial alignment between PRISM-DR’s activation maps and expert lesion annotations. This evaluation is entirely post-hoc: no lesion annotations are available during training, and the metrics are computed at inference time to test whether grade-discriminative attention coincides with pathological structure.

**Annotation source.** Lesion bounding boxes are taken from the DDR lesion-detection split, which provides Pascal VOC-format XML annotations for four lesion types [60]: microaneurysms (MA), hemorrhages (HE), hard exudates (EX), and soft exudates (SE); (757 total images).

**Pointing Game (PG).** The Pointing Game [66] measures whether the single highest-activation pixel of the CAM falls within an annotated lesion region, as defined in Equation (29). A dataset-level accuracy is obtained by averaging PG over all images. The random baseline equals the lesion area ratio: ${\mathrm{PG}}_{\mathrm{rand}}={\left|M\right|}_{1}/\left(512^{2}\right)$, which ranges from 0.09% for MA (median box width 2.5 px) to 1.62% when all lesion types are combined.

(29) $$\mathrm{PG}=1\;\left[M\left[\arg\max\mathrm{CAM}\right]=1\right].$$

**Pointing Game with tolerance (PG+tol).** A relaxed variant scores a hit if argmax(CAM) falls within d=15 px (Euclidean, via morphological dilation with a disc kernel of radius *d*) of any annotated pixel (Equation (30)). This variant is motivated by the sub-pixel scale of MA boxes: at 512 × 512 resolution, a 15-px neighbourhood corresponds to approximately 30 µm at a typical fundus camera field of view which is a clinically meaningful spatial neighborhood.

(30) $${\mathrm{PG}}_{+d}=1\;\left[\mathrm{Dilate}\left(M,d\right)\left[\arg\max\mathrm{CAM}\right]=1\right].$$

**Energy Ratio Enrichment (ER×).** Energy Ratio (ER) [67] measures the fraction of total CAM probability mass located within annotated lesion regions, as defined in Equation (31). Because ER depends on the lesion area, we normalize by the area ratio $\mathrm{AR}={\left|M\right|}_{1}/\left(512^{2}\right)$ to obtain the *enrichment factor* (Equation (32)).

(31) $$\mathrm{ER}=\frac{\sum_{\left(i,j\right):\;M_{ij}=1}{\mathrm{CAM}}_{ij}}{\sum_{i,j}{\mathrm{CAM}}_{ij}}.$$

(32) $$\mathrm{ER}\times =\frac{\mathrm{ER}}{\mathrm{AR}}.$$

ER×=1 corresponds to a spatially uniform map (random baseline); values above 1 indicate super-proportional concentration on lesion regions.

**Saliency-Box IoU.** The CAM is first min-max normalised to [0,1] and then binarised at a threshold τ relative to the per-image maximum as presented in Equation (33). Saliency-Box IoU is then defined as Equation (34). We evaluate at τ∈{0.3,0.5,0.7} to test sensitivity to the threshold. The binary mask ${\hat{C}}_{0.5}$ is also used directly in visualisation grids to demarcate the model’s foreground region without relying on a continuous colour scale.

(33) $${\hat{C}}_{\tau }=1\left[{\mathrm{CAM}}_{\mathrm{norm}}\geq \tau \right].$$

(34) $${\mathrm{IoU}}_{\tau }=\frac{|{\hat{C}}_{\tau }\cap M|}{|{\hat{C}}_{\tau }\cup M|}.$$

**FAF Gate Ratio.** This metric is specific to the FAF high-frequency gate $G\in {\left[0.1,\;1.0\right]}^{H\times W}$ (a sigmoid output trained with grade-level labels, not derived from gradients). It quantifies whether the gate preferentially retains high-frequency content at lesion locations:

(35) $$\mathrm{GR}=\frac{{\bar{G}}_{\mathrm{in}}}{{\bar{G}}_{\mathrm{out}}},\;{\bar{G}}_{\mathrm{in}}=\frac{\sum_{\left(i,j\right):\;M_{ij}=1}G_{ij}}{{\left|M\right|}_{1}},\;{\bar{G}}_{\mathrm{out}}=\frac{\sum_{\left(i,j\right):\;M_{ij}=0}G_{ij}}{512^{2}-{\left|M\right|}_{1}}.$$

GR>1 indicates that the FAF gate retains more high-frequency energy inside annotated lesion regions than outside, providing direct evidence for anatomical plausibility of the gating mechanism.

**Spearman rank correlations.** To assess grade-dependence, we compute the Spearman rank correlation $\rho_{S}$ between each per-image metric value and the image’s DR grade label, reporting the two-sided *p*-value. Two complementary correlations are tested: ER× vs. grade (expected negative: lesion area grows with grade, compressing the enrichment ratio) and PG vs. grade (expected positive: larger lesion area at higher grades increases the probability of a random hit, a measurement artefact).

### 4.5. Implementation Details

#### Architecture and Configuration

Table 5 lists the key architectural hyperparameters used in PRISM-DR and Table 6 summarizes the full training setup.

**Layer-wise Learning Rate Decay (LLRD).** To avoid degrading the pretrained ConvNeXtV2 features, separate learning rate groups are assigned to backbone, neck, and head parameter sets. The backbone receives a 0.1× multiplier (effective LR $=1.5\times 10^{-5}$), the neck (RecurrentBiFPN, FAF, MS-CLS Reasoner) receives 0.5× (=$7.5\times 10^{-5}$), and all prediction heads receive the full base LR (=$1.5\times 10^{-4}$). All groups follow the same cosine-warmup schedule shape, normalized per group by its initial LR.

**Class Weights.** To address the strong class imbalance in the combined EyePACS + DDR training corpus (approximately G0: 62%, G1: 6%, G2: 25%, G3: 2%, G4: 5%), cross-entropy is computed with per-class weights derived from sqrt-damped inverse class frequency. Because a DistributedWeightedSampler already equalizes batch-level class frequency, the weights are halved to prevent double-amplification, yielding [1.00, 1.62, 0.91, 2.69, 2.22] for grades 0–4, respectively.

### 4.6. Loop Count in the RecurrentBiFPN

The RecurrentBiFPN (See Section 3.3) shares all trainable weights across iterations; the sole parameter scaling with *T* is the per-loop positional embedding, one 256-dimensional row per loop). The parameter delta between T=2 and T=4 is 512 scalars that represents less than 0.001% of total model parameters (89.53 M). Loop count therefore controls representational depth and inference FLOPs rather than model capacity.

We set T=3 based on two observations. First, a pilot run with T=4 on the FSMT-2 training split triggered early stopping at epoch 18 due to a widening train/val loss gap that did not recover with learning rate reduction; the run was abandoned before the phased curriculum reached the auxiliary and prototype objectives at epoch 45, indicating a training stability failure at this depth. Second, the broader literature on iterative feature pyramid refinement consistently shows that a single bidirectional pass (T=1) is suboptimal, while gains plateau or reverse between T=3 and T=5 depending on dataset scale [68,69,70]. Weight-tied recurrent depth further offers the efficiency advantages documented for universal and deep equilibrium architectures [52,71], where shared-weight iteration matches or exceeds stacked parameterization at equal depth. We did not run fully converged, protocol-matched experiments for T=2 to isolate its performance; the present ablation is therefore qualitative for the *T* comparison and quantitative only for T=3.

#### 4.6.1. Phased Loss Curriculum

Training employs a four-phase loss curriculum that introduces each objective progressively, preventing early gradient conflicts between randomly initialized heads and the pretrained backbone. With E=300 total epochs, the phase boundaries are: warm-up end at epoch 20, CORAL pre-phase end at epoch 45 (=⌊0.15×300⌋), and full-weight epoch at epoch 99 (=max(45+30,⌊0.33×300⌋)). Table 7 details each phase.

The CORAL loss is detached from backbone gradients (coral_detach_backbone=true) throughout the entire training procedure. This is a permanent architectural constraint, not a phase-limited warm-up: the CORAL head shapes only its own projection layer (coral_pre: Linear 256→512) and threshold biases, while the backbone is updated exclusively through the CE, prototype, and consistency objectives. The isolation was adopted after empirical evaluation showed that allowing CORAL gradients to reach the backbone (even at reduced weight $\lambda_{\mathrm{coral}}=0.3$ with gradient clipping at norm 1.0) produced persistent validation QWK degradation relative to the detached configuration. The curvature mismatch between softmax cross-entropy and per-threshold binary cross-entropy generates conflicting gradient directions in the shared backbone parameters that clipping dampens but does not eliminate. The ordinal signal reaches the backbone indirectly through: (i) severity-weighted class penalties in the CE loss, whose weights increase monotonically with ICDR grade; and (ii) the prototype contrastive loss, which enforces grade-indexed cluster separation in the embedding space that the CORAL threshold geometry depends on.

The prototype memory momentum β follows a linear schedule from 0.99 (epoch 0) to 0.999 (epoch 300), allowing faster prototype adaptation early in training when class representations are unstable, and converging to near-stationary centroids by the final epochs. Prototype memory is updated using both global and local crop embeddings.

#### 4.6.2. Compute Environment

All experiments are conducted on a server equipped with 3x NVIDIA H200 GPUs (141 GB HBM3 each), a 224-core CPU, and shared-memory (/dev/shm) dataloader staging. Training uses DistributedDataParallel across GPUs with BF16 automatic mixed precision, leveraging H200 native BF16 tensor cores. DataLoader workers are set to 32, with images prefetched to shared memory to reduce I/O latency. A complete 300-epoch run on the combined EyePACS + DDR training set (approximately 38,000 images) requires approximately 18–24 h of wall-clock time.

## 5. Results

This section reports the performance of the proposed PRISM-DR model under in-domain evaluation and cross-dataset generalization, using the FSMT-1 and FSMT-2 protocols. In addition to PRISM-DR, we evaluate three foundation baselines, namely RETFound, RETFound-Green, and MedGemma-4B, to contextualize performance under domain shift.

### 5.1. In-Domain Performance and Cross-Domain Robustness

Table 8 reports PRISM-DR trained on APTOS2019 and evaluated both in-domain and across five external datasets. These results indicate that PRISM-DR effectively learns DR-relevant visual patterns under the source distribution, preserving ordinal severity structure while maintaining competitive discrimination.

Despite this strong source-domain performance, PRISM-DR exhibits a clear generalization gap when evaluated on external datasets. Under FSMT-1, QWK ranges from 0.4243 on EyePACS to 0.7767 on IDRiD, while macro-F1 remains substantially lower, ranging from 0.2899 on DDR to 0.3887 on IDRiD. This indicates that cross-dataset distribution shift remains a major source of performance degradation. Differences in camera characteristics, image quality, field-of-view, preprocessing pipelines, annotation conventions, and disease prevalence likely contribute to this erosion in performance.

Table 9 reports the FSMT-2 setting, where PRISM-DR is trained on the larger and more heterogeneous EyePACS and DDR datasets. This setting substantially improves cross-dataset transfer. Compared with APTOS2019-only training, QWK increases on all shared external targets, reaching 0.8353 on IDRiD, 0.8652 on APTOS2019, 0.7204 on Messidor-2, and 0.6934 on mBRSET. AUC-PR also improves markedly, particularly on APTOS2019, Messidor-2, and mBRSET, suggesting better precision–recall behavior under shifted class distributions.

The FSMT-2 results suggest that training-domain breadth is a critical determinant of robustness. Although PRISM-DR does not reach the near-perfect in-domain saturation observed in earlier configurations, the EyePACS + DDR training regime provides a more favorable balance between source-domain performance and cross-domain generalization. In particular, the improved results on IDRiD, APTOS2019, Messidor-2, and mBRSET indicate that exposure to broader acquisition and population variability enables the model to learn representations that are less tied to a single dataset distribution.

### 5.2. Ordinal Consistency vs. Class-Imbalance Sensitivity

A recurring observation across datasets is the divergence between QWK and macro-F1. In several cases, QWK remains moderate-to-high while macro-F1 is substantially lower. For example, under FSMT-1, IDRiD reaches QWK =0.7767 but macro-F1 =0.3887, while EyePACS reaches QWK =0.4243 and macro-F1 =0.3007. This pattern suggests that a substantial fraction of errors correspond to ordinally nearby severity grades rather than large severity jumps.

From an ordinal disease-grading perspective, near-miss errors are preferable to catastrophic misclassification, such as confusing proliferative DR with no DR. However, from a screening perspective, adjacent-grade errors remain clinically relevant because referral decisions often depend on thresholded categories, especially the transition from non-referable to referable DR. Therefore, QWK alone is insufficient to characterize screening performance under domain shift.

AUC-PR provides an additional perspective by capturing precision–recall behavior under class imbalance and prevalence shift. Under FSMT-1, AUC-PR varies from 0.5515 on Messidor-2 to 0.7007 on mBRSET, while under FSMT-2 it improves to 0.6931 on IDRiD, 0.7392 on APTOS2019, 0.6991 on Messidor-2, and 0.8341 on mBRSET. These improvements indicate that the broader EyePACS + DDR training configuration not only improves ordinal agreement, but also enhances decision-centric robustness in settings where positive-class prevalence and class balance differ from the source domain.

### 5.3. Comparison with Foundation Baselines and Capacity–Robustness Trade-Offs

Comparing PRISM-DR with RETFound (Table 10), RETFound-Green (Table 11), and MedGemma-4B (Table 12) reveals distinct performance regimes. RETFound exhibits inconsistent ordinal alignment, with low QWK on several datasets despite moderate Accuracy and macro-F1. RETFound-Green substantially improves over RETFound, particularly in QWK, suggesting that representation configuration and pretraining choices materially affect ordinal DR grading performance. MedGemma-4B provides relatively stable performance across datasets, especially in QWK and macro-F1, but does not consistently dominate PRISM-DR in decision-centric metrics such as AUC-PR. Note that MedGemma was also trained on EyePACS dataset [10].

Against RETFound, PRISM-DR achieves substantially stronger ordinal agreement and discrimination across all datasets in the FSMT-2 setting. Compared with RETFound-Green, PRISM-DR also achieves higher QWK on all datasets except mBRSET when evaluated in the EyePACS + DDR configuration, and it provides consistently stronger AUC and AUC-PR. Compared with MedGemma-4B, PRISM-DR achieves higher QWK on IDRiD, APTOS2019, DDR, and EyePACS, while MedGemma-4B remains stronger on Messidor-2 and mBRSET in terms of QWK. However, PRISM-DR achieves higher AUC-PR than MedGemma-4B on every dataset in the FSMT-2 evaluation, including substantial gains on DDR and EyePACS. This indicates that PRISM-DR provides a favorable robustness–capacity trade-off, particularly for decision-centric screening metrics, while MedGemma-4B offers strong ordinal stability at substantially larger model capacity.

### 5.4. Interpretability Analysis

#### 5.4.1. Qualitative Per-Grade GradCAM Patterns

**Figure 5:**
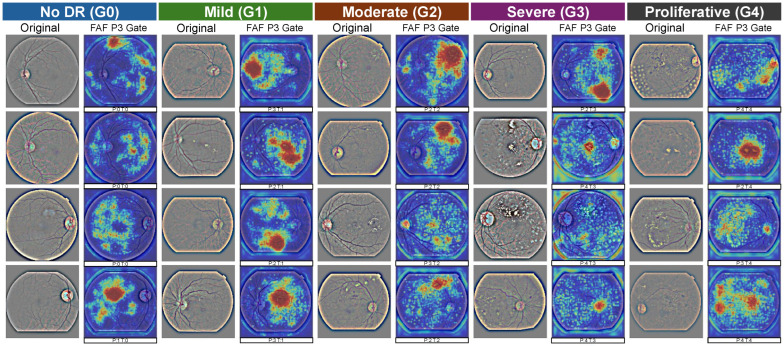
Per-grade GradCAM facegrid for PRISM-DR evaluated on APTOS2019 (FSMT-2 model, unseen test set). Post-FAF $P_{3}$ activations are shown; warm colours indicate high class-discriminative weight.

**Figure 6:**
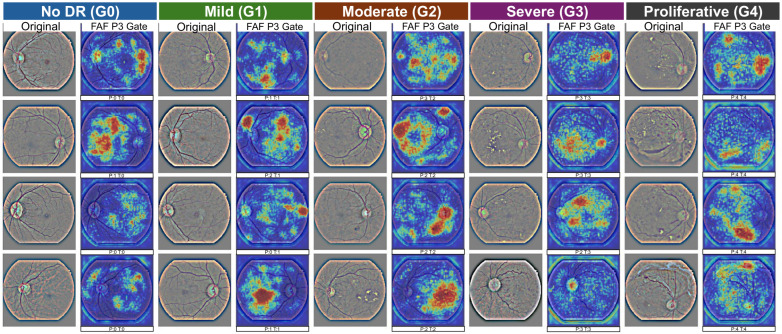
Per-grade GradCAM facegrid for PRISM-DR evaluated on IDRiD (FSMT-2 model, unseen test set). Post-FAF $P_{3}$ activations are shown. warm colours indicate high class-discriminative weight.

Figure 5 and Figure 6 show per-grade GradCAM facegrids for the FSMT-2 model evaluated on the APTOS2019 and IDRiD test sets, respectively. Each cell pairs the original fundus image with its post-FAF $P_{3}$ class-discriminative activation overlay (JET colourmap, warm = high weight).

On APTOS2019, the grade-progression of attention is broadly preserved. Grade 0 maps are predominantly cool and diffuse; two of four images show a minor focal warm patch near the optic disc, likely reflecting the model’s general sensitivity to the disc as a structural anchor rather than a DR-specific lesion response. Grades 1 and 2 exhibit increasing concentration of warm activations in the macular and vascular branching zones, consistent with early microaneurysm and hard-exudate clustering. Grade 3 maps show multi-focal high-intensity activations distributed across the peripheral retina in three of four images, and Grade 4 maps are dominated by strong disc-adjacent foci. The overall consistency between APTOS2019 attention patterns and the EyePACS + DDR in-domain evaluation aligns with the strong quantitative transfer performance (QWK =0.865, AUC-PR =0.739), demonstrating that the LSA and Ben Graham preprocessing together reduce the residual distribution gap sufficiently for the feature pyramid to operate in a similar lesion-detection regime. We explore these results more deeply in the next section.

On IDRiD, the global grade-progression trend holds but with notably higher intra-grade heterogeneity. Grade 0 maps remain mostly diffuse; Grade 1 responses are more concentrated than in APTOS, with sharp macular-region foci consistent with early microaneurysm clustering. Grade 2 presents a striking split were two images produce well-concentrated focal activations in hemorrhage-prone zones, while the remaining two yield nearly flat, low-amplitude maps. This pattern is diagnostic of a *confidence stratification* effect: the model produces clinically coherent attention on cases it classifies with high certainty but falls back to diffuse maps on ambiguous presentations. Grade 3 shows multi-focal wide-field activations in three of four images, consistent with the expected all-quadrant hemorrhagic pattern. Grade 4 maps span a range from strong disc-and-arcade activations to near-uniform cool maps, the latter consistent with proliferative cases in which fibrovascular membranes have largely replaced disc architecture.

These cross-domain patterns support the conclusion that the qualitative shift from diffuse-to-focal attention with increasing severity is a stable, transferable property of the PRISM-DR representation. However, qualitative evaluation alone cannot rule out coincidental alignment between attention and lesion locations; the following subsection provides the quantitative validation required to substantiate the claim.

#### 5.4.2. Quantitative Validation Against DDR Lesion Annotations

To move beyond qualitative inspection, we evaluate the spatial alignment between PRISM-DR’s internal activation maps and expert-annotated lesion bounding boxes. The evaluation covers all 755 images for which both a stored fundus image and at least one annotated lesion are available, and considers two maps: the post-FAF $P_{3}$ GradCAM (gradient-weighted, class-discriminative) and the FAF $P_{3}$ high-frequency gate (learned, not gradient-based). Four lesion types are annotated: microaneurysms (MA), hemorrhages (HE), hard exudates (EX), and soft exudates (SE).

**Figure 7:**
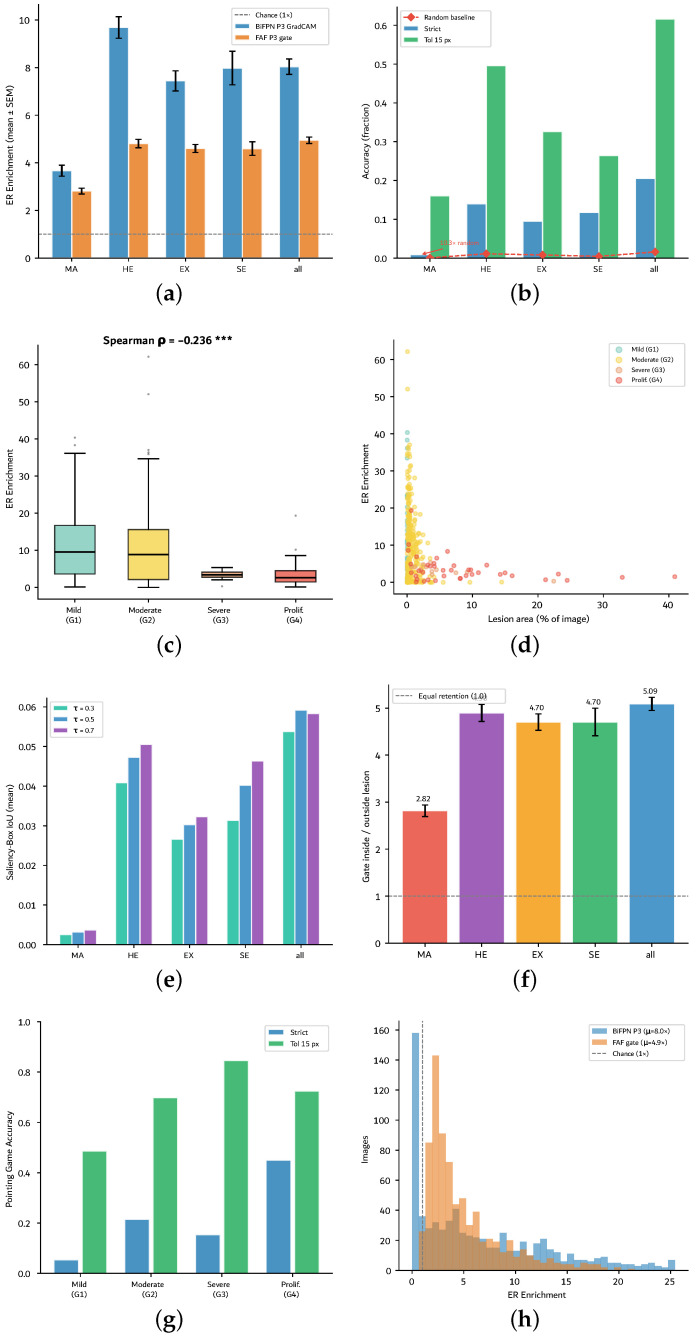
Complementary views of the quantitative alignment results, each probing a distinct aspect of the spatial correspondence between PRISM-DR’s activation maps and expert-annotated lesion regions. (**a**) Energy Ratio Enrichment by Lesion Type; (**b**) Pointing Game vs. Random Baseline; (**c**) ER Enrichment vs. DR Grade; (**d**) ER Enrichment vs. Lesion Area; (**e**) Saliency-Box IoU at varying thresholds; (**f**) FAF Gate Retention: Inside vs. Outside; (**g**) Pointing Game vs. DR Grade; (**h**) ER Distribution: P3 vs. FAF Gate; *** indicates statistical significance at *p* < 0.001.

Table 13 reports all five metrics per lesion type for both maps. Figure 7 shows the aggregate results as bar charts, box plots per DR grade, and the Spearman correlation summary.

The Energy Ratio Enrichment is the metric most directly suited to weakly-supervised interpretability evaluation because it asks a probabilistic question: *“is the attention distribution biased toward lesion regions, and by how much?"* without requiring precise localization. The BiFPN $P_{3}$ GradCAM achieves 8.04× enrichment across all lesion types, meaning the map places eight times more probability mass on annotated lesion regions than a spatially uniform map would. Per-lesion results show that the enrichment is highest for hemorrhages (9.69×), consistent with HE being the largest and most visually distinct lesion at the $P_{3}$ resolution. Soft exudates (8.0×) and hard exudates (7.4×) follow closely, while microaneurysms score lower (3.67×) because the sub-pixel scale of individual MA bounding boxes limits how much absolute energy can accumulate within them even when the model attends correctly.

The strict PG for MA (0.009) warrants careful interpretation. The random baseline for MA is 0.0009, that is a bounding box of median width 2.5 px in a 512 × 512 image covers 0.09% of pixels, so the probability of a uniformly random argmax landing inside it is 0.09%. The observed PG of 0.009 is therefore 10.3× above the random baseline, not a near-zero result. The strict-to-tolerance gap further quantifies this: PG rises 18× (from 0.009 to 0.160) when the neighbourhood is relaxed to 15 px, confirming that the model’s peak activation is spatially near microaneurysm locations without landing precisely on their tiny bounding boxes. This behavior is consistent with the expected response of a classification model, which must integrate diffuse signal from the vascular neighborhood rather than pinpoint a single 2-pixel lesion.

The bar chart (Figure 7a) confirms that the BiFPN $P_{3}$ GradCAM achieves consistently higher ER enrichment than the FAF gate across all four lesion types, with hemorrhages (HE) producing the strongest signal (9.69× vs. 4.81×). Both maps substantially exceed the chance baseline of 1.0× for every lesion category, with the GradCAM showing the largest margin for HE and SE and the smallest for MA.

The strict PG (blue) and tolerance PG (green, +15 px, presented in Figure 7b, are plotted together with the area-ratio random baseline (red diamond markers). The tolerance variant reveals the practical localization quality most clearly: across all lesion types combined, the model’s peak activation falls within 15 px of an annotated lesion in 61.6% of images, compared to a random expectation of 1.62%. The strict-to-tolerance gap is most dramatic for MA, where the 18-fold increase (0.009 to 0.160) quantifies how tightly concentrated MA activations are in spatial proximity to their tiny boxes without landing precisely inside them. This is a pattern consistent with a classification model integrating diffuse vascular neighborhood signal rather than pinpointing individual two-pixel lesions.

Figure 7c shows a negative Spearman correlation between ER enrichment and DR grade (ρ=−0.236, p<0.001). This may appear paradoxical: *“should a model not show higher lesion-aligned attention at higher severity grades?"*. The key is the area ratio: at Grade 1, lesion boxes cover ≈0.015% of the image; at Grade 4 they cover ≈5.6%. As annotated lesion area grows with severity, ER enrichment is geometrically bounded and even a map that perfectly covers all lesions cannot achieve the same ratio as when lesions are rare. Crucially, the reverse metric (strict PG) increases with grade (ρ=+0.199, p=0.0001), and this is itself a measurement artefact: larger lesion areas at higher grades increase the probability of a random hit. Both observations are therefore consistent with the model attending correctly to lesion-bearing regions across all grades; neither direction of correlation is, by itself, evidence of improvement or degradation.

Figure 7d shows that the grade-coloured scatter makes the geometric compression effect visually explicit: Grade 1 images (yellow) cluster at the left with small lesion area and high ER values spanning 0–60×, while Grade3–4 images (pink/orange) occupy the right with large lesion area and ER bounded below 10×. The hyperbolic envelope of the scatter confirms that the negative Spearman correlation is entirely attributable to this area-ratio normalization effect and not to any reduction in the model’s lesion-alignment quality at higher grades.

The Saliency-Box IoU values (0.059 for GradCAM, 0.083 for the gate), presented in Figure 7e, are substantially below those reported for detection-trained models (typically 0.3–0.6). This is expected: a classification model produces a spatially distributed activation that integrates evidence from all lesions simultaneously, rather than tightly segmenting individual boxes. The metric profiles confirm this: IoU varies by less than 0.006 across τ∈{0.3,0.5,0.7} for both maps demonstrating that the maps have a smooth gradient distribution rather than a sharp bimodal structure, and that the IoU conclusion is robust to the exact threshold choice.

The gate ratio plot presented in Figure 7f provides the most architecturally specific evidence: the FAF gate retains 2.82× more high-frequency content at MA locations, rising to 4.70× for HE, 4.70× for EX, and 5.09× across all lesion types combined. Unlike GradCAM, the FAF gate is not conditioned on a specific class and is not derived from gradients, it is a purely learned, sigmoid-bounded spatial mask trained under grade-level supervision alone. The fact that it preferentially retains high-frequency energy at annotated lesion locations therefore constitutes independent, gradient-free evidence that the frequency gating mechanism encodes anatomically plausible spatial priors without any pixel-level annotation signal.

In contrast to the negative ER–grade correlation, the Pointing Game (Figure 7g) shows a positive grade correlation ($\rho_{\mathrm{strict}}=+0.199$, p<0.001). The tolerance PG rises from approximately 0.45 at Grade 1 to above 0.85 at Grade 4 (Proliferative). As discussed, this positive slope is itself a measurement artifact: larger annotated lesion area at higher grades increases the probability of a random hit, so the metric naturally improves with grade independent of model quality. Both the negative ER correlation and the positive PG correlation are therefore internally consistent with a model that attends correctly to lesion regions across all severity levels.

The BiFPN $P_{3}$ GradCAM and the FAF $P_{3}$ gate measure fundamentally different quantities as we present in Figure 7h. GradCAM is the gradient-weighted activation of the class-discriminative layer: it shows which spatial regions contributed most to the grade prediction. The FAF gate is a learned, sigmoid-bounded spatial mask (a∈[0.1,1.0]) that the model uses to retain high-frequency content; it is not conditioned on a specific class and not derived from gradients. Their differing metric profiles are therefore informative. GradCAM achieves higher ER enrichment (8.0× vs. 5.0×) because it is explicitly optimized to be discriminative for the predicted grade. The FAF gate achieves higher IoU (0.083 vs. 0.059) and higher PG (0.311 vs. 0.205) because it produces a more spatially concentrated, binary-like signal that overlaps more tightly with individual bounding boxes. The gate also provides the direct anatomical plausibility evidence: its mean activation is 4.42× higher inside lesion regions than outside (all lesions combined), reaching 4.30× for hemorrhages and 4.21× for hard exudates. This confirms that the gating mechanism learns, without any pixel-level supervision, to preserve high-frequency content preferentially where pathological structures reside.

**Figure 8:**
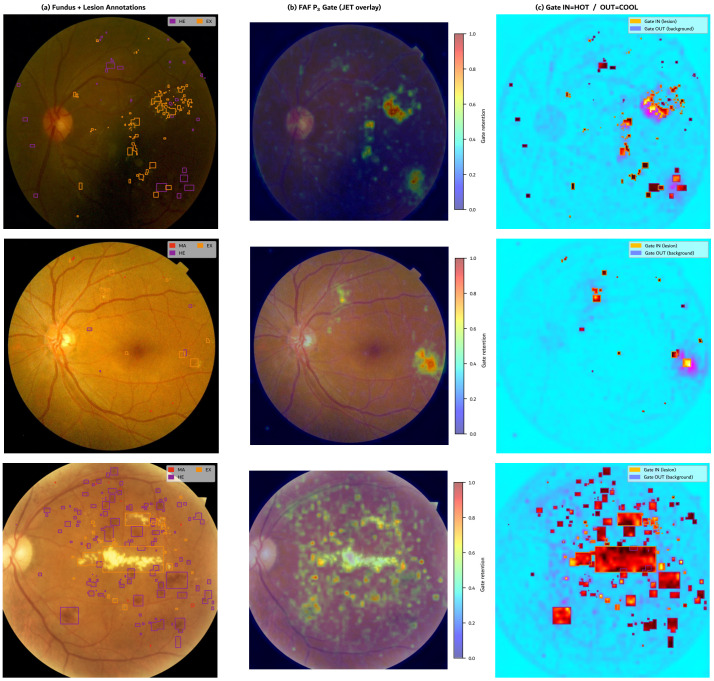
FAF $P_{3}$ gate retention visualized against DDR lesion annotations. Each row is one image; columns are: (**a**) original fundus with lesion bounding-box contours (MA = red, HE = purple, EX = orange, SE = green); (**b**) FAF gate as JET overlay; (**c**) gate split by annotation: HOT colourmap inside lesion regions, COOL outside. Strong HOT regions inside annotated boxes with surrounding COOL confirm that the gate preferentially retains high-frequency content at pathological locations.

Figure 8 shows a sample of images from the DDR annotated dataset, each presented as a three-panel strip: (i) the original fundus image with lesion bounding-box contours drawn in lesion-type colors; (ii) the FAF $P_{3}$ gate rendered as a JET heatmap overlay; and (iii) the gate split by annotation: pixels *inside* annotated lesion regions are rendered in the HOT colourmap (black → red → yellow), pixels *outside* in the COOL colourmap (dark blue → cyan). This visualization directly addresses the explicit foreground/background demarcation by making the gate’s spatial concentration at lesion locations immediately visible without relying on threshold choices or gradient computations.

Finally, these results demonstrate that the spatial alignment between PRISM-DR and expert lesion annotations is (i) consistent across lesion types, (ii) robust to metric choice, (iii) statistically significant, (iv) interpretable in terms of the geometric constraints of each metric, and (v) present independently in both a gradient-based discriminative map and a learned non-gradient gating map. The apparent paradox of opposite grade-correlation directions for ER and PG resolves cleanly once the lesion area growth with grade is accounted for, and both observations are fully consistent with correct lesion attention at all severity levels.

### 5.5. Implications and Remaining Challenges

Overall, these results support three conclusions. First, PRISM-DR learns clinically meaningful lesion evidence and preserves ordinal DR severity structure under matched source-domain conditions. Second, cross-dataset testing confirms that dataset shift remains a major barrier to reliable deployment, especially when the model is trained on a narrower source distribution. Third, training on broader and more heterogeneous datasets, as in the EyePACS + DDR FSMT-2 setting, substantially improves both ordinal agreement and precision–recall behavior across external targets.

At the same time, the results also show that no single metric fully captures screening reliability. QWK reflects ordinal consistency, macro-F1 captures class-balanced classification behavior, and AUC-PR is particularly sensitive to prevalence shift and clinically relevant positive-class detection. Therefore, robustness evaluation for DR screening should jointly consider ordinal agreement, class-balanced performance, and precision–recall behavior. Future improvements will likely require broader multi-source training, domain-aware augmentation or normalization, and objectives that explicitly align ordinal severity modeling with thresholded clinical referral decisions.

### 5.6. Limitations of Prism-DR

The CORAL ordinal head is trained with gradients isolated from the backbone (coral_detach_backbone=True), meaning the ordinal threshold loss does not directly update the primary feature extractor. The backbone is influenced by the ordinal scale only indirectly, through severity-weighted cross-entropy penalties and the prototype contrastive objective. This partitioning was a necessary response to training instability caused by curvature mismatch between the CE and per-threshold binary cross-entropy losses operating on shared parameters: the composite gradient norm exceeded 17 under full backpropagation, and gradient clipping did not recover validation QWK. As a consequence, the CORAL head functions as an ordinal calibrator applied to backbone embeddings shaped primarily by discriminative objectives, rather than a fully end-to-end ordinal learning pathway.

Despite substantial improvement under the FSMT-2 training regime, a non-trivial performance gap persists on some external targets. The effect is most pronounced on datasets with narrow class distributions, unusual acquisition conditions, or device characteristics absent from the EyePACS + DDR training mixture, most notably mobile-captured mBRSET and the small, high-quality clinical cohort IDRiD. Domain shift in fundus photography remains an open problem that architectural choices and learned normalization (LSA, BGT) can mitigate but not eliminate. The systematic divergence observed between QWK and decision-centric metrics (macro-F1, AUC-PR) under distribution shift further highlights that ordinal consistency alone does not guarantee screening-critical reliability: models that concentrate errors at adjacent-grade boundaries can still underperform at the non-referable to referable DR threshold, which is the operationally decisive decision boundary in population screening programs.

The FSMT protocol fixes training domains a priori and evaluates multi-target transfer. This reduces computational cost and directly probes single- and dual-source generalization, but does not exhaustively characterize performance under all possible training–target pairings, nor does it correspond to the leave-one-domain-out regime used in GDRBench-style benchmarks. Comparisons with methods evaluated under different protocols should therefore be interpreted with caution.

The quantitative interpretability evaluation provides multi-metric evidence that PRISM-DR’s spatial attention is non-trivially aligned with pathological retinal structure. However, the DDR annotations used are bounding boxes rather than pixel-level segmentation masks, which limits the precision of IoU-based metrics and means that attention concentrated near but not inside a box is not credited. The evaluation is also restricted to the DDR lesion-detection split and does not cover the full diversity of lesion morphologies and acquisition conditions present across all six datasets. Validation against pixel-level lesion segmentations would provide a more stringent spatial alignment test, and correlation with expert gaze data during grading would offer an independent behavioral reference not dependent on annotation format.

The choice of T=3 bidirectional fusion passes in the Recurrent BiFPN reflects an empirically constrained decision rather than a fully validated ablation. A pilot run at T=4 triggered early stopping at epoch18 due to training instability; a T=2 configuration was stable but produced consistently lower validation QWK. No protocol-matched, fully converged experiments were conducted for either alternative under the FSMT-2 curriculum, so the T=3 setting is supported by pilot evidence and literature on iterative feature pyramid refinement [68,69,70] rather than a systematic sweep. A controlled ablation over T∈1,2,3,4 remains as future work.

As discussed in the context of the FAF module, resizing fundus images to 512 × 512 imposes a resolution floor below which individual microaneurysm instances are not spatially resolvable. At this resolution, the median MA bounding box spans approximately 2.5 pixels; individual lesions are therefore below the single-feature-map-pixel threshold at $P_{3}$ (128×128, stride 4), meaning that the model cannot directly localize them but can respond to the vascular neighborhood texture associated with MA clustering. The quantitative interpretability results confirm this: the 10.3× above-chance strict Pointing Game accuracy and 3.67× Energy Ratio Enrichment for MA reflect sensitivity to the spatial context surrounding microaneurysm-bearing regions rather than direct sub-pixel lesion detection. This distinction has clinical implications: the model’s MA-related activations are evidence of lesion-associated vascular change at the neighborhood scale, not individual lesion identification. Higher input resolutions (e.g., 768×768 or 1024×1024) would reduce this limitation but at substantially increased memory and compute cost; the impact on downstream grading accuracy and spatial alignment metrics under such configurations was not evaluated in this study.

## 6. Conclusions

This work introduced PRISM-DR, a multi-objective framework for five-grade diabetic retinopathy grading that integrates multi-scale lesion modeling, ordinal-calibrated prediction, and domain-robust representation learning within a shared inference architecture. The model is designed to capture both localized retinal lesions and broader contextual patterns while preserving the ordered structure of DR severity.

PRISM-DR achieved strong in-domain performance and more importantly, when trained on the broader EyePACS + DDR cohort, it generalized well to external datasets. These results show that cross-dataset robustness depends not only on architecture, but also on the diversity and heterogeneity of the training data. Compared with RETFound, RETFound-Green, and MedGemma-4B under the same evaluation protocol, PRISM-DR provided a favorable robustness–capacity trade-off. It outperformed RETFound and RETFound-Green across all datasets in QWK and AUC-PR, and achieved higher AUC-PR than MedGemma-4B on every evaluated dataset, including large gains on EyePACS and DDR.

The qualitative GradCAM analysis further shown that PRISM-DR learns clinically meaningful attention patterns from grade-level labels alone, with activations progressing from diffuse responses in normal images to more focal lesion-related regions in higher DR grades. The interpretability evaluation substantiates the clinical coherence of the learned representations through two complementary lines of evidence. Qualitatively, multi-scale GradCAM analysis reveals that the model’s attention progresses consistently from diffuse background responses at Grade 0 to focal, anatomically plausible activations at higher severity grades, aligning with the expected spatial distribution of microaneurysms, hemorrhages, exudates, and neovascularization along the ICDR scale without any pixel-level lesion annotation at training time. Quantitatively, alignment metrics computed against 755 expert-annotated images from the DDR lesion-detection split confirm that this correspondence is statistically significant and not reducible to chance.

These results establish that the multi-scale BiFPN–FAF pipeline learns to encode lesion-relevant spatial structure as an emergent consequence of grade-level supervision alone.

## Figures and Tables

**Table 1:** The International Clinical Diabetic Retinopathy Disease Severity Scale (ICDR).

Severity Grade	Classification	Primary Clinical Indicators
Level 0	Non-apparent	Absence of retinal lesions or abnormalities.
Level 1	Mild NPDR	Presence of microaneurysms exclusively.
Level 2	Moderate NPDR	Presence of hemorrhages or microaneurysms exceeding Level 1, but falling short of Level 3 criteria.
Level 3	Severe NPDR	Meets at least one of the following criteria (4-2-1 rule): Significant intraretinal hemorrhages (>20) in all four quadrants. Confirmed venous beading in ≥2 quadrants. Obvious IRMA (intraretinal microvascular abnormalities) in ≥1 quadrant. No clinical evidence of proliferative changes (PDR).
Level 4	Proliferative (PDR)	Evidence of active neovascularization or presence of vitreous/preretinal hemorrhage.

**Table 2:** Comparison of model performance using the LODO evaluation protocol. Metrics include AUC, Accuracy (ACC), and F1-score, expressed as percentages (%). The baselines are reported as in [38,39].

	APTOS			IDRiD			Messidor		
Method	AUC	ACC	F1	AUC	ACC	F1	AUC	ACC	F1
ERM	66.4	53.2	31.6	69.6	56.5	33.9	70.6	51.3	33.7
DRGen [40]	69.4	60.7	35.7	70.8	67.7	30.6	77	64.5	37.4
Mixup [41]	65.5	49.4	30.2	70.2	64	32.6	71.5	63	32.6
MixStyle [42]	62	48.8	25	53	53.5	19.4	51.4	57.6	16.8
GREEN [43]	67.5	52.6	33.3	68.5	60.7	33	71.3	54.5	33.1
CABNet [44]	67.3	52.2	30.8	67.4	62.7	31.7	72.3	63.8	35.3
DDAIG [45]	67.4	48.7	31.6	70.2	60.2	33.4	73.5	69.1	35.6
ATS [46]	68.8	51.7	32.4	69.1	66.6	30.6	73.4	64.8	32.4
Fishr [47]	64.5	61.7	31	71.8	48	30.6	74.3	52	33.8
MDLT [48]	67.6	53.3	32.4	71.9	61.7	32.4	73.4	58.9	34.1
GDRNet [38]	69.8	52.8	35.2	72.9	70	35.1	78.1	65.7	40.5
CLIP-DR [49]	83.3	-	46.3	84.5	-	41.9	79	-	47.3
MIL-ViT [50]	80.4	68.1	46.2	85.3	40.6	39.9	81.9	60.3	42.2
GAD [39]	81.7	70.6	47	85.5	50.6	42.3	84	63.8	47.3
Dino2-DR [28]	85.6	74.84	52.21	88.33	57.69	52.63	89.24	74.32	49.17

**Table 3:** GradCAM target layers in PRISM-DR and their interpretive role.

Layer Key	Module	Captured Signal
bifpn_p3	FAF output at $P_{3}$	Fine-grained lesion cues (microaneurysms, exudate edges)
bifpn_p4	FAF output at $P_{4}$	Mid-scale hemorrhage and capillary patterns
bifpn_p5	$P_{5}$ input to MS-CLS Reasoner	Global severity context (neovascularization, disc area)
backbone_c5	Deepest ConvNeXtV2-Base stage	High-level semantic DR features
faf_p3_gate	FAF $\hat{g}$ gate at $P_{3}$	Learned high-frequency gating at fine scale
faf_p4_gate	FAF $\hat{g}$ gate at $P_{4}$	Learned high-frequency gating at mid scale

**Table 4:** Summary of publicly available datasets for DR detection and classification with 5-class distribution.

Dataset	Instances	Resolution (Min – Max)	Class Distribution
EyePACS	88,702 images 35,126 train 53,576 test	433 × 289 to 5184 × 3456	No DR: 25,810 Mild: 2443 Moderate: 5292 Severe: 873 PDR: 708
APTOS (2019)	3662 (labeled) 1928 (unlabeled)	474 × 358 to 4288 × 2848	No DR: 1805 Mild: 370 Moderate: 999 Severe: 193 PDR: 295
IDRiD	516	4288 × 2848	No DR: 134 Mild: 20 Moderate: 136 Severe: 74 PDR: 49
DDR	13,673 (755 annotations)	512 × 512 to 5184 × 3456	No DR: 6266 Mild: 613 Moderate: 4450 Severe: 602 PDR: 1023
Messidor-2	874 exams 1748 images	1440 × 960 to 2304 × 1536	No DR: 1017 Mild: 270 Moderate: 347 Severe: 75 PDR: 35
mBRSET	5164 images 1291 patients	1600 × 1600	No DR: 4124 Mild: 355 Moderate: 435 Severe: 138 PDR: 112

**Table 5:** PRISM-DR architectural configuration.

Hyperparameter	Value
Backbone	ConvNeXtV2-Base (pretrained, ImageNet-22K)
Feature pyramid channels (*C*)	256
RecurrentBiFPN loops (*T*)	3
MS-CLS Reasoner loops ($T^{\prime }$)	3
Head hidden dimension	512
Dropout rate (*p*)	0.20
CORAL gradient isolation	enabled (coral_detach_backbone=true)
Scale auxiliary heads	enabled (P3, P4, P5)
Prototype memory momentum (β)	0.99→0.999 (linear EMA schedule)
Prototype temperature	τ=0.07 (learnable)
Global crop size	512×512
Local crop size	384×384
Number of local crops	4

**Table 6:** Training hyperparameters for PRISM-DR.

Setting	Value
Total epochs	300
Batch size (per GPU)	16
Gradient accumulation steps	2 (effective batch = 32 per GPU)
Optimizer	AdamW
Base learning rate	$1.5\times 10^{-4}$
Minimum learning rate	$1\times 10^{-6}$
LR schedule	Cosine decay with linear warm-up
Warm-up epochs	20
Weight decay	0.10
Gradient clipping (max norm)	1.0
Label smoothing (ε)	0.05
AMP dtype	BF16
EMA shadow model decay	0.9999
Early stopping patience	100 epochs ($\Delta_{\min}=0.001$ QWK)
Checkpoint interval	every 20 epochs
Sampler	DistributedWeightedSampler (class-balanced)
Multi-GPU strategy	DDP (DistributedDataParallel)
Random seed	42

**Table 7:** Phased loss curriculum. λ values indicate final target weights; ramps follow a cosine schedule. QWK surrogate loss is permanently disabled ($\lambda_{\mathrm{QWK}}=0$): the soft confusion-matrix formulation does not converge to the discrete QWK metric and was found to destabilize training.

Phase	Epochs	Active Losses and Weights
1—Warm-up	0–19	CE only: $\lambda_{\mathrm{CE}}=0.50$. All other losses gated at zero. LR increases linearly from $\lambda_{\min}$ to base LR.
2—CORAL ramp	20–44	CE + CORAL cosine-ramping from ≈0.03 toward $\lambda_{\mathrm{CORAL}}=0.30$. Proto, aux, and consistency still at zero.
3—Full ramp	45–98	CE + CORAL (continuing ramp) + proto / aux / cons cosine-ramping toward their target weights: $\lambda_{\mathrm{proto}}=0.15$, $\lambda_{\mathrm{aux}}=0.10$, $\lambda_{\mathrm{cons}}=0.05$.
4—Full	99–300	All losses at final weights: $L=0.50\;L_{\mathrm{CE}}+0.30\;L_{\mathrm{CORAL}}+0.15\;L_{\mathrm{proto}}+0.10\;L_{\mathrm{aux}}+0.05\;L_{\mathrm{cons}}$.

**Table 8:** Performance of PRISM-DR model trained on the APTOS2019 dataset, reporting in-domain (*) validation (holdout test), as well as cross-dataset generalization performance using FSMT-1 validation.

Dataset	Accuracy	QWK	F1-Score	AUC	AUC-PR
APTOS *	0.8326	0.8920	0.6938	0.8953	0.6870
IDRiD	0.5846	0.7767	0.3887	0.8213	0.6365
Messidor-2	0.6490	0.5720	0.3569	0.7687	0.5515
mBRSET	0.7588	0.4725	0.3173	0.7163	0.7007
DDR	0.5864	0.5376	0.2899	0.7556	0.6204
EyePACS	0.7488	0.4243	0.3007	0.7311	0.6672

**Table 9:** Performance of PRISM-DR model trained on the EyePACS and DDR datasets, reporting in-domain (*) validation (holdout test), as well as cross-dataset generalization performance using FSMT-2 validation.

Dataset	Accuracy	QWK	F1-Score	AUC	AUC-PR
IDRiD	0.6857	0.8353	0.6186	0.8763	0.6931
Aptos2019	0.7482	0.8652	0.5191	0.8739	0.7392
Messidor-2	0.7156	0.7204	0.5499	0.8442	0.6991
mBRSET	0.8041	0.6934	0.4775	0.8716	0.8341
DDR *	0.8959	0.8607	0.7992	0.9305	0.9246
EyePACS *	0.9395	0.9199	0.8677	0.9584	0.9411

**Table 10:** Performance of the RETFound model.

Dataset	Accuracy	QWK	F1-Score	AUC	AUC-PR
IDRiD	0.3758	0.2898	0.3636	0.5964	0.3580
Aptos2019	0.6936	0.6565	0.6710	0.8604	0.6824
Messidor-2	0.4553	0.0920	0.4340	0.5218	0.4254
mBRSET	0.7418	0.0112	0.6629	0.5329	0.6236
DDR	0.5609	0.3486	0.5352	0.6801	0.5448
EyePACS	0.6960	0.0436	0.6265	0.5473	0.5937

**Table 11:** Performance of the RETFound-Green model.

Dataset	Accuracy	QWK	F1-Score	AUC	AUC-PR
IDRiD	0.5692	0.7471	0.5505	0.7073	0.4437
Aptos2019	0.7673	0.8274	0.7490	0.8451	0.6633
Messidor-2	0.5648	0.5023	0.5422	0.6071	0.4728
mBRSET	0.7952	0.5866	0.7540	0.6542	0.6817
DDR	0.6935	0.6564	0.6752	0.7435	0.5835
EyePACS	0.7387	0.4202	0.6905	0.6052	0.6185

**Table 12:** Performance of the MedGemma-4B model. Note that MedGemma was also trained on EyePACS dataset.

Dataset	Accuracy	QWK	F1-Score	AUC	AUC-PR
IDRiD	0.3824	0.7114	0.3665	0.6205	0.4021
APTOS	0.5767	0.7810	0.5559	0.7608	0.5822
Messidor-2	0.7156	0.8328	0.7223	0.7902	0.6421
mBRSET	0.8163	0.7942	0.8123	0.8015	0.7599
DDR	0.6830	0.7704	0.6859	0.7864	0.6393
EyePACS	0.7920	0.7828	0.7842	0.7772	0.7230

**Table 13:** Quantitative spatial alignment between PRISM-DR activation maps and DDR lesion-detection annotations (n=755 annotated images). PG = Pointing Game (strict); PG+tol = Pointing Game with 15-px tolerance; ER× = Energy Ratio Enrichment over area-ratio baseline; IoU@0.5 = Saliency-Box IoU at threshold τ=0.5; Gate in/out = FAF gate inside-lesion mean ÷ outside-lesion mean (gate map only). Bold entries indicate the stronger result per metric across maps. Random baseline (area ratio) indicates the expected localization accuracy obtained by random sampling, corresponding to the fraction of image area occupied by each lesion type.

Map	Lesion	PG Strict	PG +15 px	ER×	IoU@0.5	Gate in/out
BiFPN $P_{3}$ GradCAM	MA	0.009	0.160	**3.67×**	0.003	—
BiFPN $P_{3}$ GradCAM	HE	0.140	0.496	**9.69×**	0.047	—
BiFPN $P_{3}$ GradCAM	EX	0.095	0.326	**7.44×**	0.030	—
BiFPN $P_{3}$ GradCAM	SE	0.118	0.265	**7.98×**	0.040	—
BiFPN $P_{3}$ GradCAM	**All**	0.205	0.616	**8.04×**	0.059	—
FAF $P_{3}$ gate	MA	0.018	0.247	2.81×	0.008	2.55×
FAF $P_{3}$ gate	HE	0.165	0.558	4.81×	0.053	4.30×
FAF $P_{3}$ gate	EX	0.194	0.477	4.60×	0.065	4.21×
FAF $P_{3}$ gate	SE	0.155	0.315	4.60×	0.067	4.06×
FAF $P_{3}$ gate	**All**	**0.311**	**0.809**	5.0×	**0.083**	**4.42×**
*Random baseline (area ratio)*		*MA: 0.09%; HE: 1.15%; EX: 0.83%; SE: 0.47%; All: 1.62%*				

## Data Availability

This study uses exclusively publicly available, de-identified retinal fundus image datasets. No data collection involving human subjects was conducted by the authors. All datasets are accessible through the repositories listed as follows. **EyePACS** [61]: Released as part of the Kaggle Diabetic Retinopathy Detection Challenge (2015). The labeled training split (35,126 images, five-grade DR labels) is publicly available at https://www.kaggle.com/competitions/diabetic-retinopathy-detection/data (accessed on 18 August 2025). **APTOS 2019** [62]: Released for the Kaggle APTOS 2019 Blindness Detection Competition. The labeled dataset (3662 images, five-grade DR labels) is publicly available at https://www.kaggle.com/competitions/aptos2019-blindness-detection/data (accessed on 22 August 2025). **IDRiD** (Indian Diabetic Retinopathy Image Dataset) [63]: A curated clinical benchmark providing 516 high-resolution fundus images with expert-provided five-grade DR severity labels and pixel-level lesion annotations. Publicly available through the IEEE Dataport and the IDRiD Grand Challenge: https://idrid.grand-challenge.org, https://ieee-dataport.org/open-access/indian-diabetic-retinopathy-image-dataset-idrid (accessed on 10 September 2025). **DDR** (Diabetic Retinopathy Dataset) [60,72]: A large multi-center dataset comprising 13,673 color fundus images from 147 hospitals in China, with five-grade DR severity labels and image-level gradability annotations. Publicly available at https://github.com/nkicsl/DDR-dataset (accessed on 17 September 2025). **Messidor-2** [65]: A DR screening benchmark comprising 874 examinations (1748 images) from the Messidor programme. Publicly available from the ADCIS repository: https://www.adcis.net/en/third-party/messidor2 (accessed on 2 October 2025). **mBRSET** (Mobile Brazilian Retinal Dataset) [64]: A portable-camera dataset of 5164 images from 1291 patients, captured with a handheld smartphone-based fundus device (Phelcom Eyer). Published on PhysioNet with accompanying clinical and demographic metadata: https://physionet.org/content/mbrset (accessed on 14 October 2025).
